# Folate depletion induces erythroid differentiation through perturbation of de novo purine synthesis

**DOI:** 10.1126/sciadv.adj9479

**Published:** 2024-01-31

**Authors:** Adam G. Maynard, Nancy K. Pohl, Annabel P. Mueller, Boryana Petrova, Alan Y. L. Wong, Peng Wang, Andrew J. Culhane, Jeannette R. Brook, Leah M. Hirsch, Ngoc Hoang, Orville Kirkland, Tatum Braun, Sarah Ducamp, Mark D. Fleming, Hojun Li, Naama Kanarek

**Affiliations:** ^1^ Department of Pathology, Boston Children’s Hospital, Boston, MA 02115, USA.; ^2^ Graduate Program in Biological and Biomedical Sciences, Harvard Medical School, Boston, MA 02115, USA.; ^3^ Harvard School of Public Health PhD Program, Boston, MA 02115, USA.; ^4^ Harvard Medical School, Boston, MA 02115, USA.; ^5^ Harvard/MIT MD-PhD Program, Harvard Medical School, Boston, MA 02115, USA.; ^6^ Koch Institute for Integrative Cancer Research, Massachusetts Institute of Technology, Cambridge, MA 02142, USA.; ^7^ Division of Hematology/Oncology, Boston Children’s Hospital, Boston, MA 02115, USA.; ^8^ Department of Pediatric Oncology, Dana-Farber Cancer Institute, Boston, MA 02115, USA.; ^9^ Department of Pediatrics, University of California, San Diego, CA 92093, USA.; ^10^ Broad Institute of Harvard and MIT, Cambridge, MA, 02142, USA.

## Abstract

Folate, an essential vitamin, is a one-carbon acceptor and donor in key metabolic reactions. Erythroid cells harbor a unique sensitivity to folate deprivation, as revealed by the primary pathological manifestation of nutritional folate deprivation: megaloblastic anemia. To study this metabolic sensitivity, we applied mild folate depletion to human and mouse erythroid cell lines and primary murine erythroid progenitors. We show that folate depletion induces early blockade of purine synthesis and accumulation of the purine synthesis intermediate and signaling molecule, 5′-phosphoribosyl-5-aminoimidazole-4-carboxamide (AICAR), followed by enhanced heme metabolism, hemoglobin synthesis, and erythroid differentiation. This is phenocopied by inhibition of folate metabolism using the inhibitor SHIN1, and by AICAR supplementation. Mechanistically, the metabolically driven differentiation is independent of mechanistic target of rapamycin complex 1 (mTORC1) and adenosine 5′-monophosphate–activated protein kinase (AMPK) and is instead mediated by protein kinase C. Our findings suggest that folate deprivation–induced premature differentiation of erythroid progenitor cells is a molecular etiology to folate deficiency–induced anemia.

## Teaser

Folate-deprived erythroid cells activate an aberrant differentiation program that could play a role in nutritional anemia.

## INTRODUCTION

Folic acid (FA) is a vitamin required for many cellular processes, including nucleotide synthesis (*1*), methylation (*2*), and redox balance (*3*), collectively known as one-carbon (1C) metabolism (*4*). 1C metabolism requires the folate-mediated transfer of methyl or formyl groups from 1C donors, such as serine, to 1C acceptors, such as purine nucleotide intermediates (fig. S1A). Folate’s role as the 1C unit carrier is essential in all cells; however, folate is not synthesized by mammalian cells and must be consumed through the diet. Folate deficiency affects all cell types, but in postembryonic tissues, it is especially detrimental to erythroid cells. This sensitivity is demonstrated by the primary manifestation of nutritional folate deprivation—anemia (*5*).

Erythroid differentiation, or erythropoiesis, is the process that generates red blood cells from multipotent hematopoietic cells in the bone marrow. During this multistep process, progenitor cells go through several proliferation cycles to achieve abundant cell counts, while they also decrease in size, increase hemoglobin synthesis, and undergo nuclear ejection on their way to form mature erythrocytes. The differentiation program is tightly regulated and includes multifaceted cellular changes that are orchestrated mainly by the transcription factors GATA-binding factor 1 (GATA1), Kruppel-like factor 1 (KLF1), and Friend of GATA-1 (FOG1) (*6*). A notable metabolic change during erythropoiesis is the up-regulation of heme synthesis. Heme synthesis starts with condensation of mitochondrial glycine with succinyl–coenzyme A (succinyl-CoA) by the rate limiting enzyme, 5-aminolevulinic acid synthase 2 (ALAS2), to generate heme in an eight-step reaction. Glycine, which is a heme precursor, can be generated from serine in a folate-dependent reaction or through transport of extracellular glycine (*7*).

The connection between folate and erythroid differentiation was revealed in the 1940s when megaloblastic anemia was successfully treated with folate supplementation (*5*). Early studies that sought to understand the progression of folate-deficient anemia suggested DNA damage-induced apoptosis as the mechanism for reduced erythrocytic cell counts (*8*, *9*). However, this suggested mechanism does not fully support the appearance of large, hemoglobin-rich, red blood cells that are characteristic of megaloblastic anemia. This leaves the molecular etiology of nutritional megaloblastic anemia unresolved.

We investigated the molecular etiology of nutritional anemia by studying the cellular consequences of mild folate depletion in two erythrocytic cell lines: the human cell line K562 and the murine erythroleukemia (MEL) cell line. We applied metabolite profiling and biochemical assays to assess the metabolic changes and signaling events that occur following folate deprivation in both cell lines. We found that in both cell lines, mild folate deprivation induced cell differentiation in a mechanistic target of rapamycin complex 1 (mTORC1) and adenosine 5′-monophosphate–activated protein kinase (AMPK)–independent manner. We also excluded blunted RAS signaling that we detected in these cells by RNA sequencing (RNA-seq) as a key signaling for the induction of low folate-induced differentiation. However, we found that folate deprivation–induced perturbation of purine metabolism is necessary for the induction of differentiation. More specifically, the purine synthesis intermediate and signaling molecule 5′-phosphoribosyl-5-aminoimidazole-4-carboxamide (AICAR), which accumulates rapidly upon folate depletion, are necessary and sufficient for the differentiation phenotype. Moreover, we found that protein kinase C (PKC), a kinase previously demonstrated to be activated by AICAR (*10*), is necessary for the observed differentiation in cell lines and primary murine erythroid progenitor cells. We postulate a signaling axis that involves AICAR-dependent activation of PKC, which is a reported inducer of erythroid differentiation (*11**–**13*). Our results suggest a molecular etiology to folate deprivation–induced anemia: Early, aberrant progenitor differentiation can result in low red blood cell counts due to reduced number of proliferation cycles and can also explain the appearance of large, heme-filled erythrocytes, both characteristic of megaloblastic anemia that is caused by folate deprivation.

## RESULTS

### Folate depletion up-regulates heme synthesis and induces erythroid differentiation

**Fig. 1.**
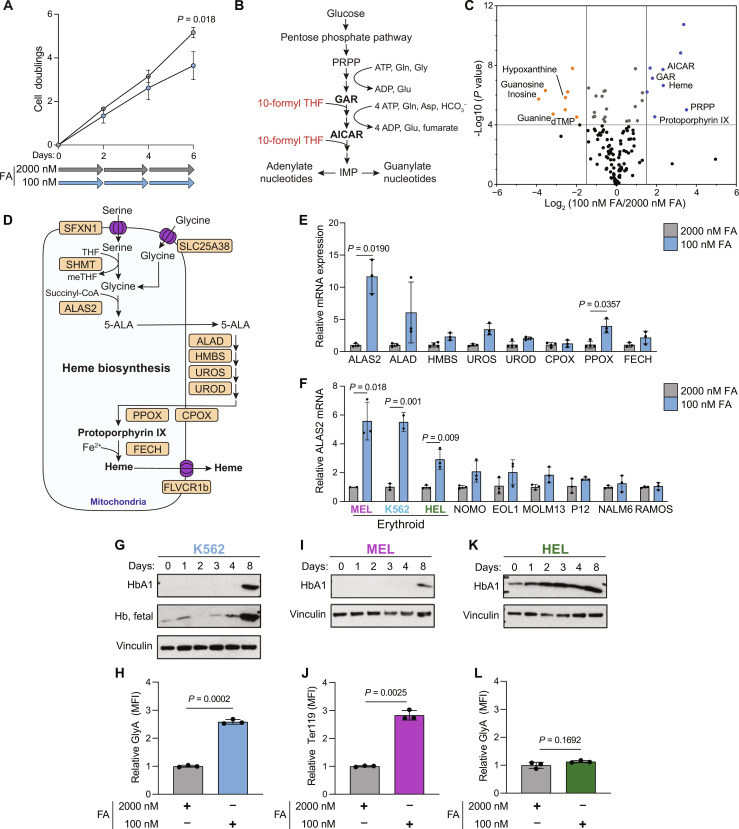
Folate depletion induces differentiation in erythroid cancer cell lines. (**A**) Experimental timeline (bottom) and proliferation (top) of K562 cultured for 6 days in folate-free RPMI supplemented with 2000 or 100 nM FA. (**B**) Simplified schematic of de novo purine synthesis. (**C**) Volcano plot depicting day 6 metabolic changes between K562 cells cultured in 2000 and 100 nM FA. PRPP, phosphoribosyl diphosphate; dTMP, deoxythymidine monophosphate. (**D**) Schematic of the human heme biosynthesis pathway. (**E**) Reverse transcription quantitative polymerase chain reaction (RT-qPCR) analysis of heme biosynthesis genes in 2000 nM or 100 nM FA. (**F**) RT-qPCR analysis of *ALAS2* in the indicated cell lines following 6 days culture in 2000 or 100 nM FA. (**G**) Western blot analysis of hemoglobin (Hb) A1 and fetal expression in K562 over 8 days in 100 nM FA. (**H**) Cell surface GlyA levels in K562 measured by flow cytometry. MFI, mean fluorescence intensity. (**I**) Western blot analysis of hemoglobin A1 in MEL. (**J**) Cell surface Ter119 levels in MEL as measured by flow cytometry. (**K**) Western blot analysis of hemoglobin A1 in HEL. (**L**) Cell surface GlyA levels in HEL as measured by flow cytometry. All data shown are means (±SD) of three biological replicates except (E) *n* = 3 to 4 and (F) *n* = 2 to 3 (K562 and MEL).

Standard mammalian cell culture medium contains supraphysiological levels of most nutrients (*14*). RPMI and Dulbecco’s Modified Eagle medium (DMEM), two commonly used cell culture medium, contain 2000 and 9000 nM FA (Gibco), respectively, while healthy adults have a plasma folate concentration around 20 to 80 nM (*15*, *16*). Cell lines in culture are well adapted to this high concentration of folate, and it is not detrimental to normal cell function. We sought to investigate the metabolic changes in erythroid cells following a mild folate depletion, rather than complete folate withdrawal, because this mild folate insufficiency is likely more similar to the stress induced by nutritional folate deprivation that does not result in full systemic depletion of folate. We used 100 nM FA and compared this to the medium standard 2000 nM FA. This concentration led to a modest proliferation defect of K562, a chronic myelogenous leukemia cell line with the potential to undergo erythroid differentiation (Fig. 1A) (*17*, *18*). Through metabolite profiling of 6-day folate-deprived K562, we observed significant accumulation of the purine synthesis intermediates 5′-phosphoribosyl-glycinamide (GAR) and AICAR (Fig. 1, B and C). These two metabolites are substrates for reactions that require 10-formyl tetrahydrofolate (THF) (Fig. 1B) and are expected to become the rate limiting steps of de novo purine synthesis upon folate depletion (*19*). Several nucleotide salvage intermediates, including hypoxanthine, inosine, and guanosine, were depleted under low FA conditions (Fig. 1C), as these can be used to regenerate inosine monophosphate (IMP) levels when purine synthesis is impaired (*20*). Unexpectedly, we also observed a significant increase in intracellular heme, as well as accumulation of the last intermediate of heme synthesis, protoporphyrin IX (Fig. 1, C and D). To test whether heme synthesis is actively enhanced in folate-depleted K562 cells, we assayed the mRNA expression of heme biosynthesis pathway genes. We found significant up-regulation of the rate-limiting (*21*) enzyme *ALAS2* (Fig. 1E). While depletion of folate increases mRNA expression of heme biosynthesis pathway components, there was no accompanied up-regulation of 1C metabolism genes (fig. S1B). The synthesis of a single heme molecule requires eight molecules of glycine that can be generated from serine through the action of the folate-dependent enzyme serine hydroxymethyltransferase (SHMT) or imported from exogenous pools. Although serine was underused under low FA conditions (fig. S1C), as expected from folate depletion, glycine levels were still sufficient to support heme synthesis due to utilization of exogenous glycine (fig. S1D). The increase in total heme was not associated with an increase in mitochondrial mass or membrane potential, which suggests a utilization of heme outside of the mitochondrial respiratory complexes (fig. S1, E and F).

Increased heme synthesis is a hallmark of the erythroid differentiation process (*22*). To test whether folate deprivation induces erythroid differentiation in additional hematopoietic cell lines, we measured induction of *ALAS2* expression in nine hematopoietic cell lines following folate deprivation. We observed up-regulation of *ALAS2* only in the three erythroid leukemia lines included in the panel [MEL, K562, and human erythroleukemia (HEL)] and not in cell lines derived from other lineages (Fig. 1F). The induction of *ALAS2* expression was not correlated with growth rate in low FA (fig. S1G). In addition, we observed no induction of the nonerythroid heme synthesis enzyme, *ALAS1* (fig. S1H). This supports an erythroid specific role of heme synthesis in folate-deprived cells as opposed to a broad up-regulation of heme synthesis in all leukemia cell lines.

We therefore validated the induction of heme synthesis and the increase in differentiation markers following mild folate deprivation in two additional erythroid cell lines; the mouse line, MEL (*23*), and the human line, HEL (*24*). Over 8 days in low FA, K562 cells increase expression of fetal hemoglobin [found in K562 cells although these are not embryonic-derived cells (*25*)] and hemoglobin A1 (Fig. 1G), as well as the erythroid cell surface marker glycophorin A (GlyA) (Fig. 1H). Both MEL and HEL increased hemoglobin A1 protein expression (Fig. 1, I and K). Despite hemoglobin levels increasing in low FA, these results are not reflecting through benzidine staining (fig. S1, I and J). We assayed the erythroid differentiation surface markers Ter119 and GlyA found on MEL and HEL, respectively, following folate deprivation. While no change in GlyA surface expression was observed in HEL, MEL significantly increased expression of Ter119 (Fig. 1, J and L). Together, our data reveal induction of erythroid differentiation by mild folate deprivation.

Our metabolite profiling data of 6-day folate-deprived K562 revealed accumulation of protoporphyrin IX, a metabolite that, together with iron, generates a complete heme molecule (Fig. 1D and fig. S2A) (*7*). This raised the possibility that folate-deprived K562 suffers from lack of iron due to increased demand from up-regulated heme synthesis. However, neither iron supplementation, either as free iron (Fe^2+^) or transferrin-bound iron (Fe^2+^-transferrin), nor iron chelation (using deferoxamine) altered proliferation (fig. S2B) or differentiation (fig. S2, C and D), although these treatments result in the expected changes in transferrin receptor expression (fig. S2E). These data rule out a role for iron availability or limitation as a driver of folate deprivation–induced differentiation.

### Induction of differentiation in response to mild folate depletion occurs independently of DNA damage

Because folate deficiency is suggested to cause anemia through DNA damage in erythroid progenitor cell and this is considered to be the cause of megaloblastic anemia (*8*, *9*, *26*), we set to monitor DNA damage in mild folate deprivation (100 nM) in K562 cells. We added lower concentrations of folate to model more severe folate depletion as controls and included 2000, 100, 50, and 20 nM FA culture conditions for 48 hours. Folate depletion at these doses and timescale allowed cell proliferation in culture, and we observed no significant impairment in proliferation (fig. S3A). We observed a significant depletion of THF in all three low FA concentrations (fig. S3B), but this has not yet manifested in a proliferation defect. We also observed a dose-dependent increase in serine and decrease in glycine (fig. S3, C and D), and accumulation of the purine synthesis intermediates GAR and AICAR (fig. S3, E and F). On the other hand, guanosine 5′-triphosphate (GTP) levels were more stable (fig. S3G). After characterizing the metabolic impact of these various FA concentrations, we set to test whether erythroid cells cultured in these FA concentrations suffer from DNA damage. We assessed the level of DNA damage via phosphorylation of the DNA damage–responsive protein checkpoint kinase 1 (Chk1) and the level of apoptosis via cleaved caspase III. We found that unlike more severe FA depletion (*27*), 100 nM dose of FA does not result in increased p-Chk1/Chk1 ratio or cleaved caspase III following 6 days of FA depletion in K562 and MEL (fig. S3, H to M). Comparison of 100 nM FA folate depletion to H_2_O_2_ treatment revealed a mild phosphorylation of Chk1, only later than 4 days of folate deprivation, and no detection of phospho-H2A histone family member X, S139 (γH2AX) in folate-deprived cells (fig. S3N). In addition, immunofluorescence quantification of γH2AX in mild or severely folate-deprived cells reveals no significant increase in DNA-damage following 2 days of culture, while known inducer of DNA damage, doxorubicin, leads to a significant increase in γH2AX in both K562 and MEL (fig. S3, O and P). These data confirm that mild folate deprivation results in erythroid differentiation independently of DNA damage. This suggests that DNA damage–induced stress response or apoptosis is not the cause of the reduced erythroid cell numbers found following mild folate deprivation.

### Purine biosynthesis inhibition is an early event following folate deprivation, and purine supplementation rescues folate depletion–induced differentiation

**Fig. 2.**
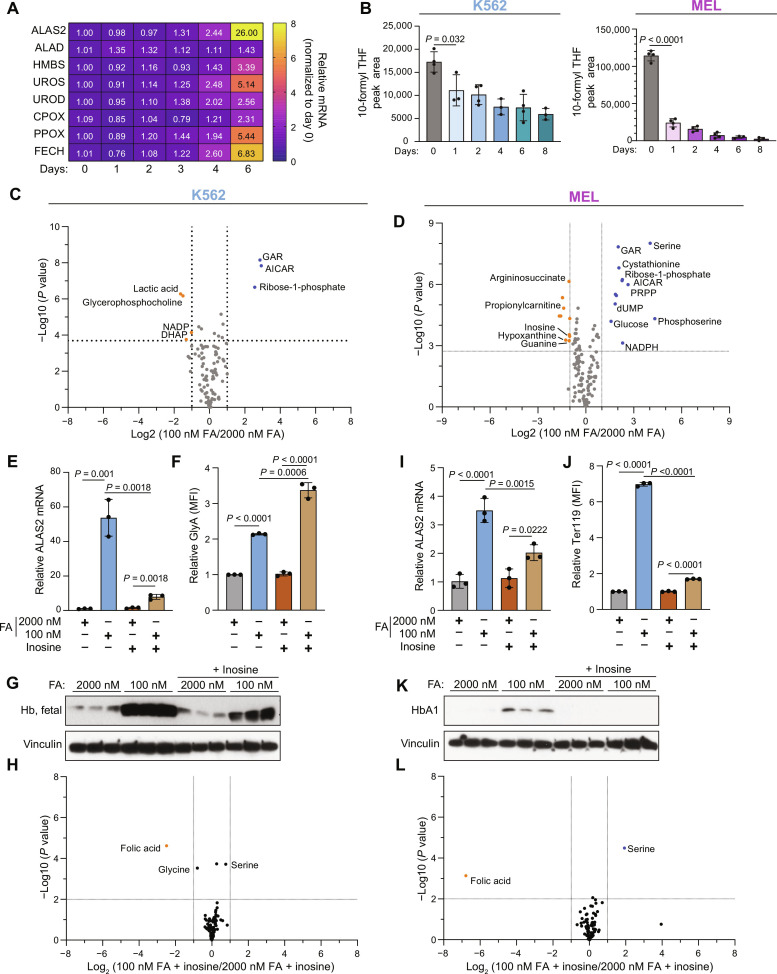
Purine synthesis is disrupted rapidly following folate deprivation, and this disruption is essential for folate deprivation–induced differentiation. (**A**) RT-qPCR analysis of heme synthesis mRNA expression in K562 over 6 days in 100 nM FA. (**B**) Total 10-formyl THF in K562 (left) and MEL (right), as measured by liquid chromatography–mass spectrometry (LC-MS). (**C** and **D**) Volcano plots depicting the metabolic changes between 2000 and 100 nM FA at day 2 in K562 (C) and MEL (D). DHAP, dihydroxyacetone phosphate; dUMP, deoxyuridine monophosphate. (**E**) RT-qPCR analysis of *ALAS2* mRNA levels in K562 following 6 days in 2000 or 100 nM FA, with or without inosine (100 μM) supplementation. (**F**) Cell surface GlyA in K562 following 6 days in 2000 or 100 nM FA with or without inosine (100 μM) supplementation as measured by flow cytometry. (**G**) Western blot analysis of hemoglobin levels following 6 days in 2000 or 100 nM FA with or without inosine supplementation (100 μM). (**H**) Metabolic changes in K562 cultured in inosine supplemented with 100 nM versus 2000 nM FA medium. (**I**) RT-qPCR analysis of *Alas2* mRNA levels in MEL following 6 days in 2000 or 100 nM FA, with or without inosine (100 μM) supplementation. (**J**) Cell surface Ter119 in MEL following 6 days in 2000 or 100 nM FA with or without inosine (100 μM) supplementation as measured by flow cytometry. (**K**) Western blot analysis of hemoglobin levels following 6 days in 2000 or 100 nM FA with or without inosine supplementation (100 μM). (**L**) Metabolic changes in MEL cultured in inosine supplemented with 100 nM versus 2000 nM FA medium. All data shown are means (±SD) of three biological replicates except (B) K562 (*n* = 3 to 4) and MEL (*n* = 4).

FA is directly and indirectly involved in many metabolic reactions, including nucleotide synthesis, amino acid metabolism, and methylation reactions. Therefore, we asked which metabolic changes in folate-deprived erythroid cells drive differentiation. We addressed this by first mapping the kinetics of the cellular response to low folate availability; transcriptionally, induction of heme biosynthesis gene expression in K562 occurs at day 4 following folate deprivation (Fig. 2A). Heme levels were increased following these gene expression changes at day 6 (fig. S4A). These transcriptional and metabolic effects followed the depletion of 10-formyl THF as early as 1 day following folate depletion (Fig. 2B). Metabolically, the purine synthesis pathway (fig. S4B) was the first pathway to show significant perturbations in both K562 and MEL cells as early as day 2 following folate deprivation (Fig. 2, C and D): At day 2, K562 and MEL cells had 7 and 21 significantly altered metabolites, respectively, with AICAR, GAR, and ribose-1-phosphate (R1P), the only shared metabolites between the two cell lines. AICAR and GAR are intermediates of the purine synthesis pathway, and R1P is the byproduct of purine salvage reactions (Fig. 1B and fig. S4B). Although folate is essential for IMP and deoxythymidine monophosphate (dTMP) synthesis, examination of nucleotide levels in K562 and MEL showed that while there is a slight reduction in adenosine 5′-triphosphate (ATP) and GTP in K562 and MEL over 4 days of FA depletion, no major pyrimidine synthesis inhibition occurs (fig. S4, C and D). Therefore, although pyrimidine synthesis inhibition can be associated with myeloid leukemia differentiation (*28*), it is not likely to be the metabolic driver of the erythroid differentiation we observe here. In addition, the ratio of glutathione/oxidized glutathione does not change significantly, and the reduced form of nicotinamide adenine dinucleotide phosphate (NADP^+^) (NADPH)/NADP and reduced form of nicotinamide adenine dinucleotide (oxidized form) (NAD^+^) (NADH)/NAD ratios are significantly higher in K562 after 8 days in 100 nM FA. These data suggest that folate depletion is not associated with increased reactive oxygen species (ROS) or redox imbalance (fig. S4E).

Previous studies in erythroleukemia have shown that both purine supplementation (*29*, *30*) and inhibition of purine synthesis (*31*, *32*) can induce erythroid differentiation. Therefore, the early inhibition of purine synthesis led us to hypothesize that perturbation of this pathway drives a stress response that results in erythroid cell differentiation. To assess this hypothesis, we rescued purine synthesis by supplementation with inosine (100 μM), a purine salvage intermediate, that enables purine synthesis while bypassing folate-dependent de novo synthesis (fig. S4B). Inosine supplementation restored the proliferation rate of K562 and MEL cells in low FA (fig. S4, F and G). There was no effect of inosine on proliferation in high FA–cultured cells. Inosine supplementation also blocked folate deprivation–induced differentiation, as observed by the lack of induction of *ALAS2* expression (Fig. 2, E and I), the reduction in hemoglobin synthesis (Fig. 2, G and K), and the reduction in the erythroid surface marker Ter119 in MEL cells (Fig. 2J). However, GlyA surface expression was not rescued with inosine in K562 (Fig. 2F). In addition, treatment with inosine erased nearly all the metabolic changes associated with low FA at day 2 in K562 and MEL cells leaving only FA, serine, and glycine significantly different (Fig. 2, H and L). As expected, inosine treatment rescued de novo purine synthesis defects and nucleotide levels (fig. S4, G to K). This suggests that neither the absence of FA nor the accumulation of intracellular serine drives differentiation on their own but that perturbation of purine synthesis is the driver of folate deprivation–induced differentiation.

These data (Fig. 2, H and L) may also indicate that inosine supplementation under low FA conditions allows limiting pools of folate molecules to be redirected to other nonpurine, folate-dependent reactions so that low FA medium supplemented with inosine may permit cells to maintain all cellular folate–required reactions and avoid the metabolic stress that is otherwise associated with folate deprivation. Further work will be needed to address this specific question.

### AICAR accumulation is sufficient to drive differentiation

**Fig. 3.**
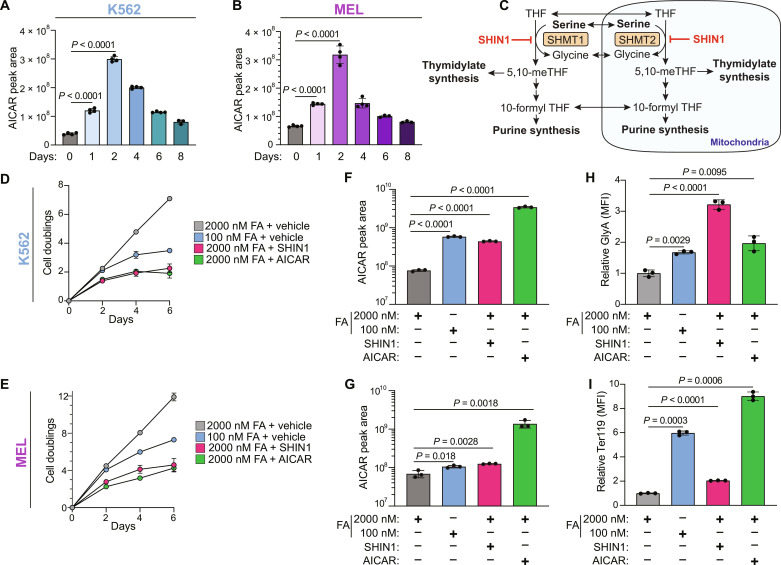
1C metabolism inhibition and AICAR supplementation induce erythroid differentiation. (**A** and **B**) AICAR peak area in (A) K562 and (B) MEL following 8 days in 100 nM FA as measured by LC-MS. (**C**) Schematic depicting the target of SHMT inhibitor, SHIN1. (**D** and **E**) Cell proliferation of (D) K562 and (E) MEL over 6 days following treatment with vehicle, 100 nM FA, SHIN1 (1.25 μM), or AICAR (500 nM). (**F** and **G**) Intracellular AICAR levels in (F) K562 and (G) MEL following 2-day treatment with vehicle, 100 nM FA, SHIN1 (1.25 μM), or AICAR (500 nM) as measured by LC-MS. (**H**) Induction of differentiation, as measured by GlyA expression in K562 following 6-day treatment with vehicle, 100 nM FA, SHIN1 (1.25 μM), or AICAR (500 nM). (**I**) Induction of differentiation, as measured by Ter119 expression in MEL following 6-day treatment with vehicle, 100 nM FA, SHIN1 (1.25 μM), or AICAR (500 nM). All data shown are means (±SD) of three biological replicates except [(A) and (B)] *n* = 4.

The early metabolic changes following folate deprivation included an increase in AICAR levels (Fig. 3, A and B). We therefore applied two additional approaches to phenocopying this metabolic change: AICAR supplementation and the 1C metabolism inhibitor, SHIN1 (*33*). SHIN1 inhibits the enzymes SHMT1 and SHMT2 and lowers intracellular levels of reduced folate, thereby mimicking nutritional folate deprivation and increasing AICAR levels (Fig. 3C). We used 500 μM AICAR and 1.25 μM SHIN1, doses that slowed proliferation rate but did not induce a cytostatic or cytotoxic effect in K562 or MEL (Fig. 3, D and E). Compared to folate deprivation, AICAR supplementation resulted in significantly higher levels of intracellular AICAR, while SHIN1 treatment resulted in comparable AICAR accumulation (Fig. 3, F and G). Similar to cells cultured in low folate, cells were treated with either AICAR or SHIN1 differentiate, as evidenced by up-regulation of erythroid cell surface markers (Fig. 3, H and I).

### Folate depletion and 1C inhibition induce changes in gene expression related to RAS and mTOR signaling

**Fig. 4.**
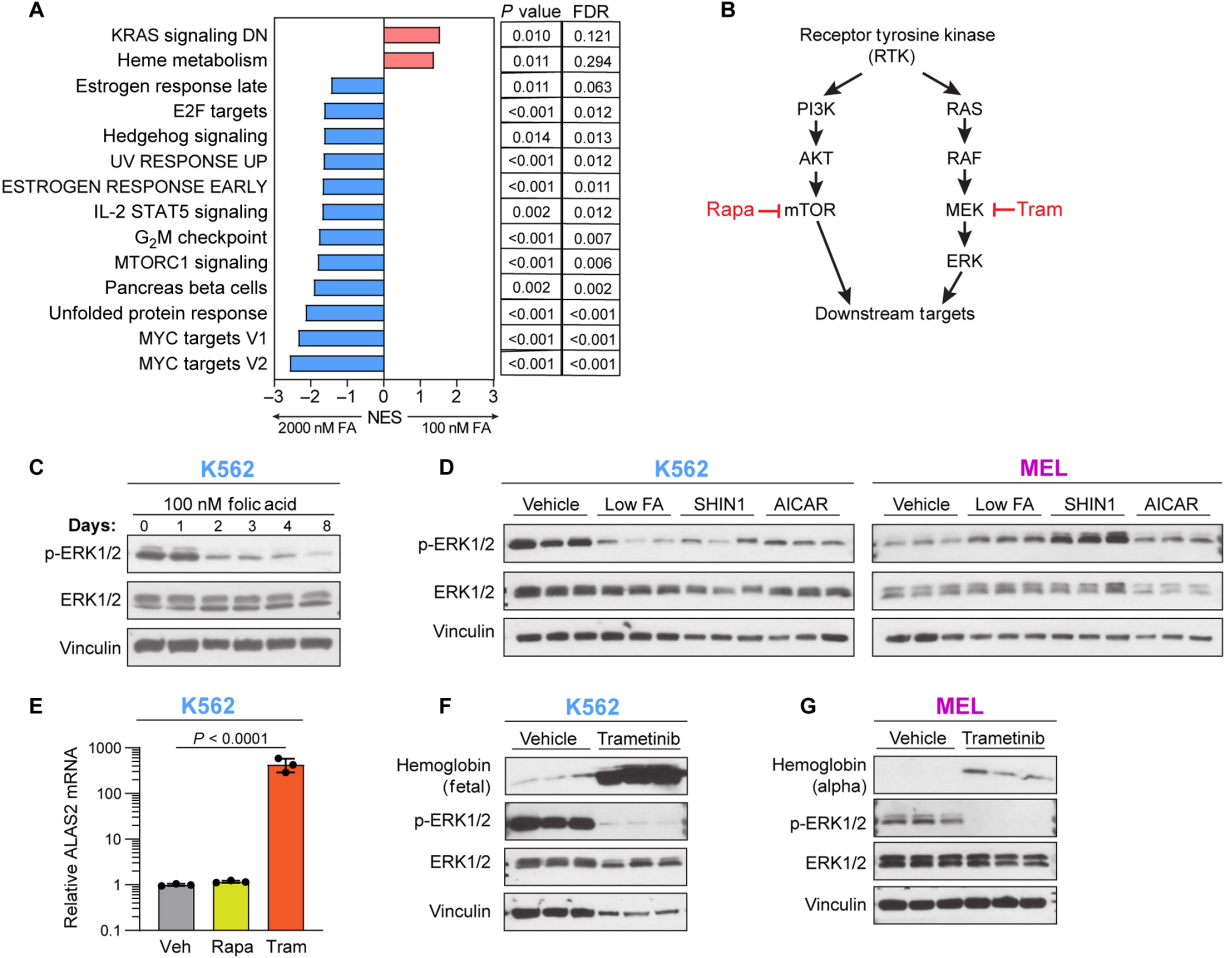
Folate depletion and 1C metabolism inhibition induce alterations to RAS signaling. (**A**) GSEA using hallmark gene sets on K562 cells in 100 or 2000 nM FA. UV, ultraviolet; IL-2, interleukin-2; STAT5, signal transducers and activators of transcription 5; FDR, false discovery rate; NES, normalized enrichment score. DN, down. (**B**) Simplified schematic of RAS signaling with two inhibitors, trametinib (tram) and rapamycin (Rapa). PI3K, phosphatidylinositol 3-kinase. (**C**) RAS signaling activity over 8 days in 100 nM FA in K562, as determined by phosphorylation of extracellular signal–regulated kinase 1/2 (ERK1/2). (**D**) RAS pathway activity in K562 and MEL following 4-day treatment with 100 nM FA, SHIN1 (1.25 μM), or AICAR (500 nM). (**E**) RT-qPCR analysis of K562 ALAS2 mRNA expression following 4-day treatment with rapamycin (100 nM) or trametinib (30 nM). (**F** and **G**) Western blotting of RAS activity and hemoglobin expression following 4-day treatment with trametinib (30 nM). All data shown are means (±SD) of three biological replicates.

Our metabolic characterization of K562 and MEL revealed a timeline of the metabolic changes following folate depletion and suggested a direct connection between purine nucleotides and the induction of a transcriptional erythroid differentiation response (Fig. 2A). To better understand the transcriptional changes that result from folate depletion and induce differentiation, we performed RNA-seq on K562 and MEL 3 days following folate depletion, SHIN1 treatment, or AICAR supplementation. This time point is before the transcriptional changes associated with erythroid differentiation (Fig. 2A) and immediately following the spike in AICAR (Fig. 3, A and B). Principal components analysis (PCA) was used to cluster the K562 and MEL samples by the RNA-seq data (fig. S5, A and B). As with our metabolomics and cell proliferation data, transcriptomic profiling revealed similar profiles between folate depletion, SHIN1 treatment, and AICAR supplementation as observed by the overlapping clusters. While folate depletion and SHIN1 were almost completely overlapping, AICAR displayed a degree of separation, especially in MEL (fig. S5B). This further supports the mild nature of our folate depletion and SHIN1 treatment–induced differentiation. Differential gene expression analysis of K562 and MEL revealed 750 and 125 significant differentially expressed genes (DEGs) in low FA (100 nM FA) compared to vehicle (2000 nM FA), respectively (fig. S5C). Most of these DEGs are shared between low FA and SHIN1. AICAR induced more DEGs in K562 and MEL, 844 and 527, of which many are unique to AICAR treatment. Together, low FA, SHIN1, and AICAR all induce differentiation in K562 and MEL, but the early transcriptional profile of AICAR supplementation is unique. We performed gene set enrichment analysis (GSEA) to identify significantly enriched, or depleted, pathways under our treatment conditions. GSEA on K562 in low folate revealed an enrichment in heme metabolism and a decrease in KRAS and mTORC1 signaling (Fig. 4A; genes down-regulated by KRAS are depicted as transcriptionally increased in 100 nM FA). Heme metabolism is a GATA1-dependent gene set that is an erythroid differentiation hallmark (*34*), and its early induction further suggests that the differentiation process is already beginning at this early time point following folate depletion.

### RAS inactivation is sufficient but not necessary for induction of erythroid differentiation

The top scored pathway in our transcriptional analysis of K562 in low FA is KRAS signaling in the downward direction (Fig. 4A). Reduction in expression of KRAS genes also scored highly in K562 following SHIN1 treatment and in MEL following low FA (fig. S5, D and E). The connection between RAS and folate depletion stood out as an attractive pathway to investigate for two reasons. First, KRAS pathway (Fig. 4B) activity is regulated by the purine guanosine triphosphate, GTP. RAS-GTP leads to active signaling, while RAS–guanosine diphosphate leads to pathway inactivation. We have shown that folate depletion leads to decreased GTP and guanylate nucleotide levels (fig. S4, C and D). Second, previous work has found evidence that KRAS pathway inhibition can affect differentiation (*35*, *36*). Folate depletion resulted in reduced RAS pathway activity in K562 as early as day 2 (Fig. 4C). In addition, SHIN1 and AICAR treatment both reduced RAS signaling in K562; however, MEL had the opposite response and increased RAS activity in response to folate depletion, SHIN1 treatment, and AICAR supplementation (Fig. 4D). The explanation for active RAS signaling in response to folate depletion is unclear, as is the lack of continuity with the RNA-seq data. Although K562 and MEL diverged in their RAS activity following folate depletion, chemical inhibition of mitogen-activated protein kinase kinase (MEK), a downstream target of RAS, by trametinib (Fig. 4B) induced differentiation in both K562 and MEL as indicated by *ALAS2* mRNA and hemoglobin levels (Fig. 4, E to G). Together, these data suggest that while down-regulation of the RAS pathway is sufficient to drive differentiation, RAS signaling down-regulation is not necessary for folate depletion–induced differentiation, at least in MEL.

The GSEA analysis provided no evidence of DNA damage response following low FA, SHIN1, or AICAR treatment. The DNA repair gene set was down-regulated in low folate in MEL. To confirm this result, we assessed γH2AX levels after 4-day treatment with low FA, SHIN1, or AICAR in K562 and MEL (fig. S5, F and G). We did not observe any evidence of DNA damage at this time point, which corroborated our previous finding that folate depletion–induced differentiation is independent of a DNA damage response (fig. S3).

### mTORC1 inactivation is not necessary or sufficient to induce erythroid differentiation

Depletion of cellular purine levels inhibits mTORC1 activation (Fig. 4A) (*37*). However, while purines were significantly depleted in K562 cells at early time points (fig. S4C), we observed only a mild depletion in MEL at the early time points (fig. S4D). Accordingly, we found a decrease in mTORC1 activation in K562 but to a lesser extent in MEL (fig. S6A). Further, perturbation of the mTORC1 signaling pathway by the inhibitors Torin1 and rapamycin under high FA conditions did not induce differentiation of K562 and MEL cells (Fig. 4E and fig. S6, B to E), ruling out sufficiency of mTORC1 inactivation for driving folate deprivation–induced erythroid differentiation. This negative result was not driven by strong inhibition of proliferation, similar to the MEK inhibitor trametinib (fig. S6F).

### AMPK activation is not necessary or sufficient to induce erythroid differentiation

**Fig. 5.**
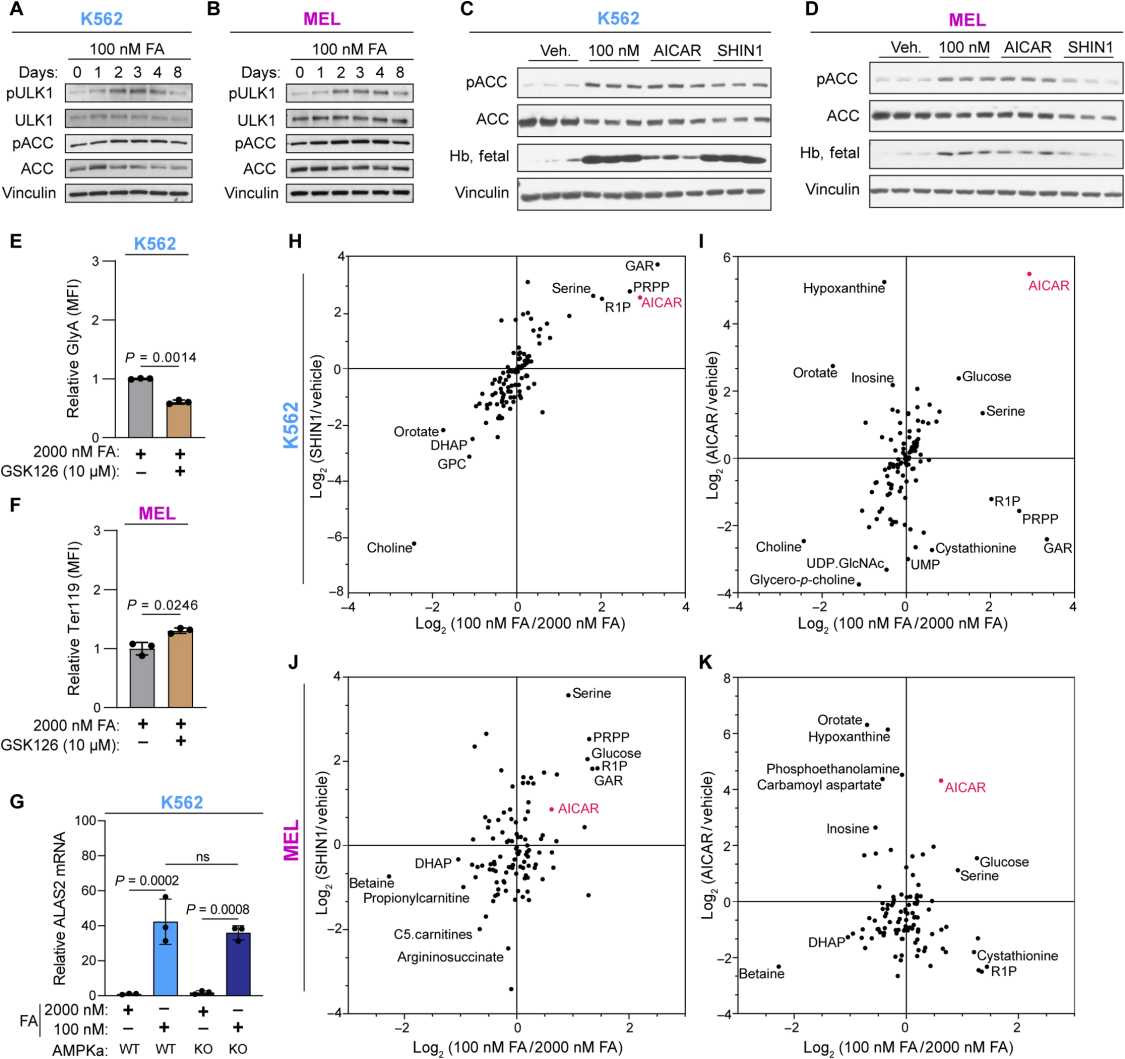
AMPK is not necessary, nor sufficient, to induce erythroid differentiation. (**A** and **B**) Western blot analysis of AMPK signaling in K562 (A) and MEL (B) over 8-day culture in 100 nM FA as determined by phosphorylation of ACC and ULK1. (**C** and **D**) Western blot analysis of AMPK signaling and hemoglobin expression in (C) K562 and (D) MEL following 6-day treatment with vehicle, 100 nM FA, SHIN1 (1.25 μM), or AICAR (500 nM). (**E** and **F**) Cell surface GlyA or Ter119 expression as measured by flow cytometry in (E) K562 and (F) MEL following treatment with the AMPK agonist, GSK126 (10 μM). (**G**) RT-qPCR analysis of *ALAS2* mRNA expression in AMPKa1/a2 wild-type (WT) and DKO K562 cells cultured in 2000 or 100 nM FA for 6 days. (**H** to **K**) Comparison of the fold change of 100 nM/2000 nM FA versus SHIN1/vehicle [(H) and (J)] and AICAR/vehicle versus 100 nM/2000 nM [(I) and (K)], in K562 (I) and MEL [(J) and (K)]. UDP, uridine 5′-diphosphate; GlcNAc, *N*-acetylglucosamine; ns, not significant. All data shown are means (±SD) of three biological replicates.

Unlike the somewhat inconsistent purine depletion between the two cell lines, the levels of the AMPK activator AICAR (*38*) increased significantly as early as 1 day following folate deprivation in both K562 and MEL cells (Fig. 3, A and B), and AICAR alone was sufficient to induce erythroid differentiation (Fig. 3). AICAR accumulation, as well as other intermediate metabolites of the purine synthesis pathway (fig. S7, A and B), occurs early enough to be acting as an inducer of the differentiation program. AMPK signaling was activated shortly after the elevation in AICAR levels as measured by the phosphorylation of acetyl-coA carboxylase (ACC) and Unc-51-like autophagy-activating kinase 1 (ULK1), two principle downstream targets of AMPK (Fig. 5, A and B) (*39*). In addition, treatment of K562 and MEL with low FA, SHIN1, or AICAR increased both AMPK signaling and hemoglobin expression (Fig. 5, C and D). To investigate whether AMPK signaling is sufficient to induce erythroid differentiation, we applied the pharmacologic AMPK activator GSK621 (*40*). While this compound did not compromise the proliferation rate of K562 and MEL cells (fig. S7, C and E) and it activated AMPK (fig. S7, D and F), it did not induce differentiation in K562 and MEL cells (Fig. 5, E and F). Further, when we tested whether AMPK signaling is necessary for folate depletion–induced differentiation by genetically targeting both *AMPKa1* and *AMPKa2* [*AMPKa* double knockout (DKO)] (fig. S7, G and H), we observed no blunting of *ALAS2* expression following folate deprivation (Fig. 5G), indicating that AMPK signaling is not necessary for induction of the differentiation program. In sum, although AMPK is activated in folate-deprived erythroid cells, it is neither sufficient nor necessary for folate depletion–induced differentiation.

This result led us to revisit the role of AICAR in the induction of differentiation downstream of folate deprivation; in K562, each of the perturbations that results in high AICAR levels—low FA, AICAR, and SHIN1—induces erythroid differentiation (Fig. 3) but, unexpectedly, not through AMPK (Fig. 5, E to G). We therefore asked whether a change in another metabolite is shared by these conditions and is the inducer of the differentiation. To better compare the metabolic profiles of SHIN1 and AICAR treatment on K562, we compared the log_2_ fold change between low FA (100 nM FA) and both SHIN1 and AICAR in both K562 and MEL (Fig. 5, H to K). While low FA and SHIN1 induced a very similar metabolic response, the comparison of low FA and AICAR is metabolically different. We found that AICAR is one of the sole metabolites changing positively in each cell line and treatment condition. In MEL cells, low FA and AICAR, but not SHIN1, activated AMPK (Fig. 5D). This can be the result of the accumulation of metabolites other than AICAR in the SHIN1-treated MEL cells; one of these metabolites is glucose—an upstream suppressor of AMPK signaling (Fig. 5, I and J). These data not only confirmed the central role played by AICAR but also revealed that additional cellular events that ensue under conditions of 1C metabolism blockade are necessary for the induction of differentiation. The fact that AICAR supplementation alone induced differentiation is fascinating and implies that the AICAR-induced cellular response likely includes some downstream events that are beyond AMPK activation and essential for the metabolically induced erythroid differentiation.

### PKC is necessary for folate depletion–induced differentiation

**Fig. 6.**
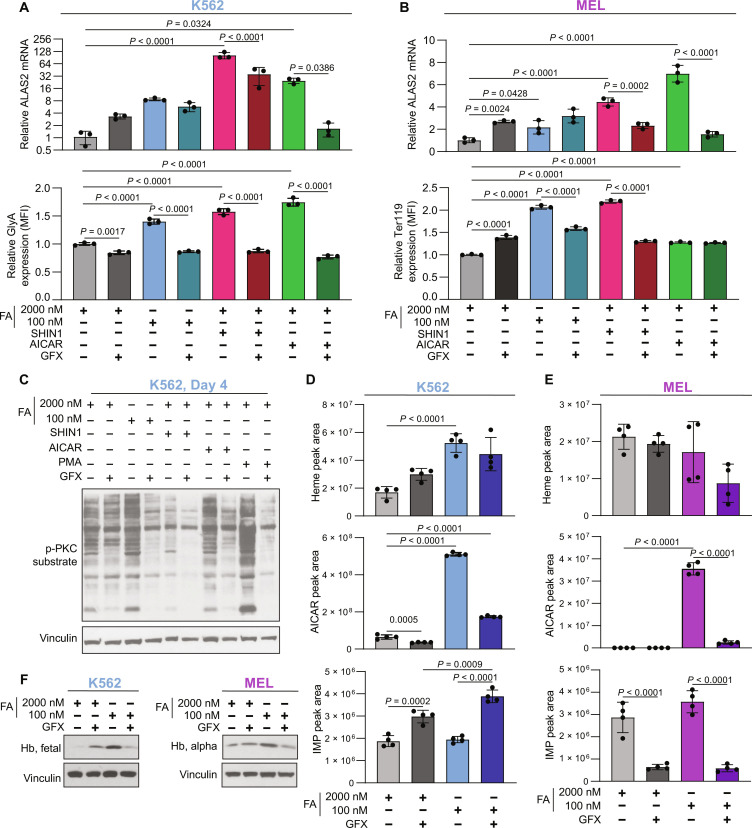
PKC activation is necessary for inducing erythroid differentiation. (**A**) RT-qPCR analysis of *ALAS2* mRNA expression and surface GlyA following 6-day treatment with low FA, SHIN1, or AICAR, in combination with the PKC inhibitor, GFX (5 μM) in K562. (**B**) RT-qPCR analysis of *Alas2* mRNA expression and surface Ter119 following 6-day treatment with low FA, SHIN1, or AICAR, in combination with GFX in MEL. (**C**) Western blot analysis of PKC signaling in K562 cells treated with low FA, SHIN1 (1.25 μM), or AICAR (500 nM) treatment plus PKC inhibition with GFX (5uM) for 4 days. (**D** and **E**) Selected metabolite abundance following 6-day culture of (D) K562 or (E) MEL in 2000 or 100 nM FA plus GFX (5 μM). (**F**) Western blot analysis of hemoglobin as treated in (D) and (E). All data shown are means (±SD) of three biological replicates except [(D) and (E)] *n* = 4.

In addition to its role in activating AMPK, AICAR can activate PKC (*41*), and PKC inhibition rescues AICAR-induced growth inhibition (*10*). Because PKC is a reported inducer of erythroid differentiation (*11**–**13*), we hypothesized that AICAR induces erythroid differentiation through PKC activation. We therefore investigated the role of PKC in mediating folate depletion–induced differentiation. First, we tested the necessity of PKC signaling in folate deprivation–induced differentiation by inhibiting PKC signaling. We treated K562 and MEL with the PKC inhibitor GF109203X (GFX), alone or in combination with low FA, SHIN1, or AICAR. We first assessed the toxicity of the treatment through its impact on cell proliferation. In both K562 and MEL, GFX alone compromised proliferation over 6 days of treatment (fig. S8, A and B). As expected, treatment with low FA, SHIN1, and AICAR impaired proliferation. The addition of PKC inhibition by GFX rescued AICAR-induced proliferation effect in K562 and MEL. However, GFX treatment resulted in the opposite effect in folate-deprived or SHIN1-treated cells. The mechanism driving this difference between the treatments combinations is unclear, but we suspect that this is related to other 1C metabolism functions and not to the metabolic axis we study here that includes perturbation of 1C metabolism, purine synthesis inhibition, AICAR up-regulation, and induction of a differentiation program. PKC inhibition by GFX mitigated metabolic differentiation in both K562 and MEL, as reflected by the markers of erythroid differentiation *ALAS2* and GlyA/Ter119 (Fig. 6, A and B). This revealed the necessity of PKC signaling in mediating differentiation in response to low FA, SHIN1, and AICAR. While these data support a role for PKC signaling in mediating folate depletion–induced differentiation, there is no evidence for widespread activation of PKC signaling in response to low FA, SHIN1, or AICAR treatment (Fig. 6C). SHIN1 treatment led to a decrease in phosphorylated PKC substrates. These data suggest that PKC’s role in differentiation may be part of a specific target and signaling cascade and not due to widespread activation of PKC signaling. PKC inhibition rescued the folate deprivation–induced hemoglobin accumulation and reduced hemoglobin levels in folate-deprived K562 and MEL cells (Fig. 6F). At the metabolic level, PKC inhibition resulted in less heme accumulation and reduction in other metabolic aberrations that were caused by folate deprivation (Fig. 6, D and E). In an attempt to reveal what PKC isoform is involved in the PKC signaling downstream to folate deprivation, we assayed the phosphorylation of PKC-α/βII, PKC-θ, PKC-δ, PKC-ζ/λ, and pan-phospho-PKC, and phosphoserine PKC, as well as for PKC-ζ, PKC-δ, PKD/PKCμ, and PKCα for any upper bands, which might represent a phosphorylated protein. We analyzed PKC signaling after 16 hours in 0, 100, and 2000 nM FA, as well as treatment with the PKC activator phorbol 12-myristate 13-acetate (PMA), and the PKC inhibitor GFX, using Western blot. We observed a specific upper band that appeared in folate-deprived K562 cells (both 100 and 0 nM FA) over PKC-ζ and PKC-δ and that might reflect a phosphorylation event (fig. S8, C and D). However, this was not the case in the MEL cells, and we are not sure whether the discrepancy between the results stems from suboptimal detection of mouse PKC by the antibody or from biological difference between K562 and MEL cells. Next, we set to experimentally test the sufficiency of PKC activation with the PKC activator PMA to induce differentiation in erythroid cells. PMA worked as expected and activated PKC in both K562 and MEL cells (fig. S8, E and F). PMA induced *Alas2* expression in MEL cells (fig. S8H), suggesting that PKC activation is sufficient for induction of differentiation in these cells. However, PMA treatment resulted in cell death in K562 cells after treatment of 24 hours, rendering *ALAS2* expression and differentiation marker measurements noninformative (fig. S8G). Hence, we chose to focus on studying the effect of the PKC inhibitor, GFX in our next experiments. Together, these data suggest that PKC is sufficient in MEL cells and is necessary in K562 and MEL cells for folate deficiency–induced differentiation. However, while PKC has been reported to respond to AICAR accumulation, it is not likely to function as a direct activator of differentiation-related gene expression as suggested by the delayed induction of differentiation-related gene expression following PKC activation.

### Folate deprivation depletes choline metabolism intermediates, but choline supplementation is not sufficient to rescue folate depletion–induced differentiation

Our metabolite profiling of K562 and MEL cells treated with low FA, AICAR, and SHIN1 revealed a significant reduction in the choline metabolism pathway, including choline, glycerophosphocholine (GPC), and phosphocholine (Fig. 5, H to K, and fig. S9A). The metabolic association between choline and folate metabolism stems from a shared reaction: In the methionine cycle, the folate form 5-methyl THF is required for the conversion of homocysteine to methionine by the enzyme Methylenetetrahydrofolate reductase (MTHFR). Alternatively, methionine can be synthesized from homocysteine by the enzyme betaine-homocysteine S-methyltransferase (BHMT) using betaine, instead of 5-methyl THF, as the methyl donor. Betaine is synthesized from choline by the enzyme choline dehydrogenase (CHDH) (fig. S9A). This connection presented two possibilities: (i) Choline metabolism is up-regulated in low FA to support the methionine cycle and maintain cellular methylation, and (ii) the depletion of both FA and choline could lead to methylation defects and epigenetic reprogramming that induces differentiation. With these possibilities in mind and coupled with the fact that choline metabolism is essential for murine and human erythropoiesis (*42*), we verified reduction of metabolites of this pathway in 1C metabolism–perturbed K562 (fig. S9, B to E) and MEL (fig. S9, F to I). To test the role of choline in folate depletion–induced differentiation, we supplemented K562 with choline (500 μM) under 2000 or 100 nM FA conditions. Supplementation with choline did not alter proliferation (fig. S9J) or rescued differentiation (fig. S9K), and choline supplementation increased *ALAS2* mRNA expression in low FA compared to low FA alone. These results suggest that lack of choline is not a sufficient cause of folate deprivation–induced differentiation.

### Folate deprivation and 1C metabolism inhibition accelerate PKC-dependent differentiation of primary murine erythroid progenitors

**Fig. 7.**
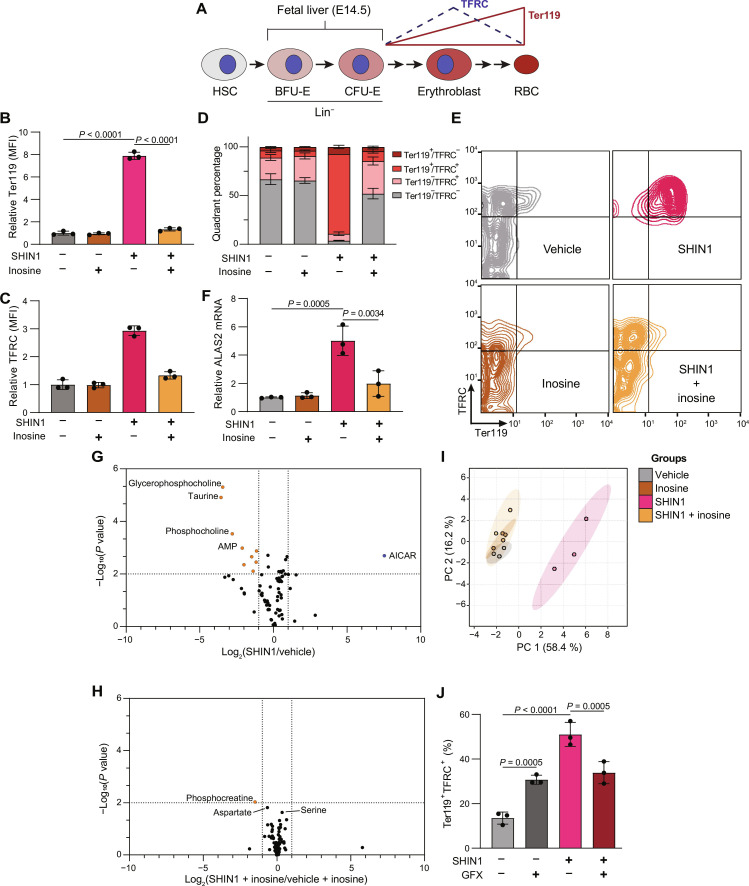
Folate deprivation induces differentiation in murine primary erythroid progenitor cells. (**A**) Schematic representing murine fetal erythropoiesis from a population of BFU-E and CFU-E progenitor cells isolated from E14.5 fetal livers. HSC, hematopoietic stem cell; RBC, red blood cell. (**B**) Cell surface Ter119 levels on erythroid progenitor cells following 4-day treatment with vehicle, SHIN1 (1.25 μM), inosine (100 μM), or SHIN1 + inosine. (**C**) Cell surface TFRC levels on erythroid progenitor cells following 4-day treatment with vehicle, SHIN1, inosine, or SHIN1 + inosine. (**D**) Flow cytometry quadrant analysis of Ter119 and TFRC expression following 4-day treatment with vehicle, SHIN1, inosine, or SHIN1 + inosine. (**E**) Representative flow cytometry plots from quantification in (D). (**F**) RT-qPCR analysis of *Alas2* mRNA expression in murine primary erythroid progenitor cells treated for 4 days with SHIN1, inosine, or SHIN1 + inosine. (**G**) Metabolic changes of vehicle- or SHIN1-treated (2 days) murine primary erythroid progenitor cells. (**H**) Metabolic changes of vehicle- or SHIN1-treated (2 days) murine primary erythroid progenitor cells supplemented with inosine. (**I**) PCA analysis of metabolite profiling data from murine primary erythroid progenitor cells with and without SHIN1 treatment and inosine supplementation. (**I**) Metabolic changes of vehicle- or SHIN1-treated (2 days) murine primary erythroid progenitor cells. (**J**) Ter119- and TFRC-positive erythroid progenitor cells following 4-day culture in vehicle, SHIN1, GFX (5 μM), or SHIN1 + GFX. Progenitor cells were cultured in SFEM II expansion medium. All data shown are means (±SD) of three biological replicates.

We next tested whether folate deprivation induces differentiation in primary erythroid progenitor cells (Fig. 7A). For this, we used an ex vivo murine erythroid progenitor differentiation system (*36*, *43*, *44*) that allows controlled nutritional and pharmacological perturbations of 1C metabolism in primary progenitor cells with an established readout of differentiation. We isolated erythroid progenitors from fetal livers of embryonic day 14.5 (E14.5) embryos and purified an enriched population of erythroid cells primarily composed of the erythroid-committed progenitor populations BFU-E (burst forming unit–erythroid) and CFU-E (colony-forming unit–erythroid) (*45*). This ex vivo differentiation model of erythroid cells can be set up as two phases, expansion and differentiation, as dictated by the presence or absence of growth factors in the medium. For addressing our biological question, we focused on culturing cells in the expansion phase to allow maintenance of undifferentiated progenitor cells, maintained by insulin-like growth factor–1 (IGF-1) and stem cell factor (SCF) that are present in the medium. The differentiation phase, achieved by removal of growth factors, is less suitable for our study because the strong differentiation drive is likely to mask any differentiation effect of 1C metabolism inhibition. The optimal commercial medium commonly used for progenitor expansion [Serum-Free Expansion Medium II (SFEM II)] contains supraphysiological levels of folate. We therefore cultured the purified erythroid population in a custom folate-depleted RPMI-based expansion medium that contains additional growth factors. Notably, erythroid progenitor cells cultured in this custom medium supplemented with 2000 nM FA proliferate successfully but trend toward an early differentiation phenotype (fig. S10A). Because we wanted to test whether folate depletion induces differentiation of erythroid progenitor cells, our custom folate-free RPMI-based expansion medium was unsuitable, and we shifted to solely using SFEM II expansion medium with SHIN1 as our means to perturb 1C metabolism. SFEM II expansion medium maintained ~60% of progenitor cells in an undifferentiated, Ter119^−^/CD71^−^ cell state over 4 days of culture (fig. S10A). SHIN1 treatment of primary progenitor cells inhibits cell growth after 4 days, and this can be rescued by inosine supplementation (fig. S10B). Further, SHIN1 treatment induced differentiation that is rescued by inosine supplementation (Fig. 7, B to F). Comparison of vehicle- to SHIN1-treated erythroid progenitors indicated that inosine supplementation rescued nearly all metabolic changes associated with SHIN1 treatment (Fig. 7, G to I). As seen in K562 and MEL, AICAR increased following SHIN1 treatment and was the only significantly accumulated metabolite (Fig. 7G). The accumulation of AICAR following SHIN1 that is rescued with inosine treatment and the reduced levels of 5-methyl THF and serine in SHIN1-treated progenitor cells that are not rescued by inosine supplementation (fig. S10, C and D) further corroborate the role of purine synthesis impairment in regulating differentiation, as opposed to signaling induced by direct sensing of the depleted folate or serine levels. Inosine supplementation significantly increased hypoxanthine levels, in agreement with the metabolic fate of inosine as a substrate for salvage purine synthesis (fig. S10E). As shown in K562 and MEL cells, SHIN1 treatment had no effect on IMP levels, and only a modest effect on nucleoside triphosphate levels that was rescued with inosine (fig. S10, F and G).

In agreement with our results in erythroid cell lines, SHIN1 treatment induced differentiation of primary erythroid progenitor cells, and this was reversed by inosine supplementation (Fig. 7, B to E). We next investigated the role of PKC signaling in our erythroid progenitor model and cotreated cells with SHIN1 and the PKC inhibitor GFX. As in the erythroid cancer cell lines K562 and MEL, PKC inhibition attenuated the differentiation induced by perturbation of 1C metabolism as determined by reduced Ter119^+^/TFRC^+^ cells [Transferrin receptor protein 1 (TFRC)] (Fig. 7J and fig. S10I). Notably, PKC inhibition compromised cell proliferation of SHIN1-treated progenitor cells while increasing proliferation as a single treatment (fig. S10H), strengthening the hypothesis that cell differentiation of 1C metabolism–perturbed cells is a cell-survival response that allows cells to maintain viability at times of metabolic stress induced by disrupted 1C metabolism.

We have demonstrated that AICAR treatment can induce erythroid differentiation in K562 and MEL and that AICAR is the sole metabolite increasing following SHIN1 treatment in primary progenitor cells. Therefore, we tested whether AICAR treatment can induce differentiation in primary progenitor cells and if PKC is necessary for this AICAR-induced differentiation; we found that AICAR alone induced differentiation in primary progenitor cells (fig. S10, J and L) but was also toxic to these cells (fig. S10K). Unexpectedly, GFX rescued AICAR-induced toxicity (fig. S10K); however, GFX treatment failed to block AICAR-induced differentiation (fig. S10, J and L).This result suggests a role for AICAR in inducing toxicity and differentiation in primary progenitor cells, and it provided further evidence that alterations to purine synthesis, through inhibition of 1C metabolism, or supplementation with purine intermediate, AICAR, can induce differentiation of primary erythroid progenitor cells.

Together, folate depletion and 1C inhibition induce differentiation in primary early erythroid progenitors. This induction of differentiation can occur even in the presence of IGF-1 and SCF and is blocked by supplementation with exogenous nucleosides and by inhibition of PKC, suggesting that purine levels are sensed and their depletion activates premature differentiation through a PKC-dependent signaling mechanism.

## DISCUSSION

Deprivation of the essential vitamin folate results in megaloblastic anemia in children and adults (*46*, *47*). This disease remains a major health incumbrance in underdeveloped countries (*48*, *49*) and in underserved populations, where nutritional shortage of folate still prevails even in our era of folate fortification and common supplementation (*16*, *50*). Megaloblastic anemia is characterized by the appearance of big, hemoglobin-filled erythrocytes (*51*, *52*). Early studies have suggested that folate-deficient anemia is a result of DNA damage–induced apoptosis (*8*, *9*). It has been thought that the absence of folate decreases thymidylate synthesis and, subsequently, the ratio of 3′-deoxythymidine 5′-triphosphate:deoxyuridine triphosphate. As a result, uracil is misincorporated into DNA, induces DNA damage, and leads to increased erythroid apoptosis, which manifests as anemia. However, although several studies have called into question this mechanism of folate-deficient anemia because of lack of molecular evidence for both DNA damage and apoptosis of erythroid progenitors (*53*, *54*), no alternative hypothesis has yet emerged.

**Fig. 8.**
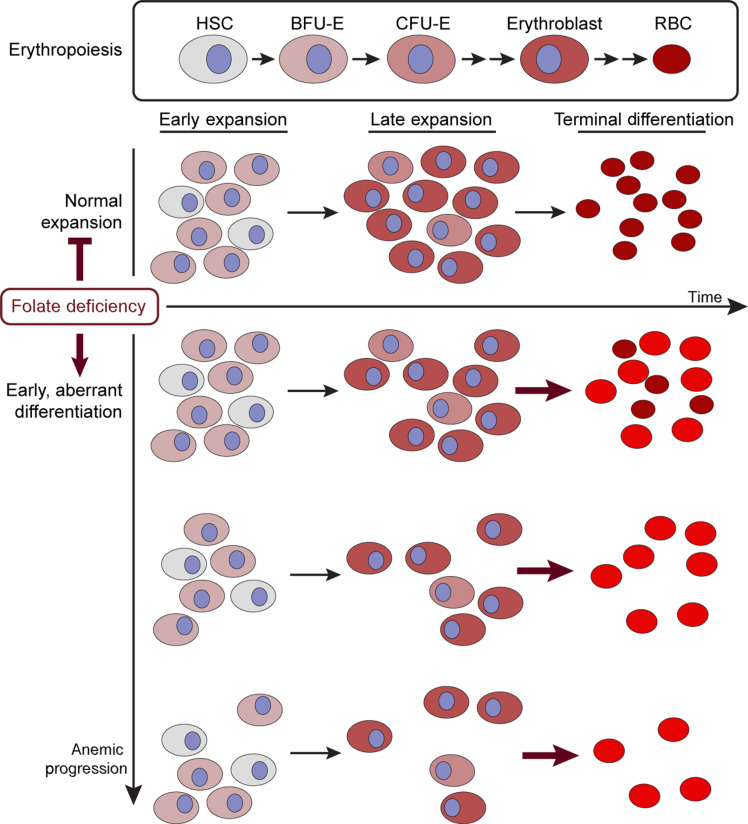
Model of folate depletion induced differentiation. Normal erythropoiesis involves commitment and expansion of hematopoietic stem cells into the erythroid lineage. Late expansion of erythroid progenitors is followed by terminal differentiation into red blood cells (**top**). Following folate depletion, we observe a premature induction of differentiation of erythroid lineage cells and an inhibition of expansion. Premature differentiation can deplete the erythroid progenitor population by preventing expansion and inducing differentiation (**bottom**). These findings subsequently recapitulate the clinical observations of folate deficiency included megaloblastic anemia.

Our data indicate that folate deprivation induces a metabolic stress that results in aberrant differentiation of erythroid cells. Premature differentiation of progenitor cells can result in insufficient number of cell division cycles that normally precede differentiation and reduced cell numbers of cells of that lineage (Fig. 8). Our results suggest an etiology to the low cell count of erythroid cells in megaloblastic anemia and to the unique manifestation of this disease—large, heme-filled cells. We describe here a folate deprivation–induced increase in heme metabolism that is regulated transcriptionally. While we have not yet identified the upstream signaling cascade that is driving the transcriptional response to folate deprivation, we postulate that the drop in cellular folate is being sensed, either directly or through purine levels, leading to signal transduction and activation of a differentiation program. Our data rule out the involvement of two major signaling pathways that respond to changes in purine levels—the mTORC1 and AMPK pathways—and we postulate that another signaling cascade bridges the drop in folate availability and activation of the differentiation program in erythroid cells.

Notably, we provide evidence that the signaling pathway bridging folate availability and activation of erythroid differentiation involves the kinase PKC. Inhibition of PKC rescues 1C metabolism perturbation–induced differentiation in K562, MEL, and primary progenitor cells, and its activation induces differentiation in MEL cells. Our results confirm previous connections between AICAR and PKC (*10*), suggesting an axis between high AICAR, PKC activation, and induction of the gene expression program that results in erythrocyte differentiation. Previous studies have presented conflicting views of PKC’s involvement in erythroid differentiation (*55*, *56*), so future work should focus on the context specific regulation of differentiation by PKC.

By mimicking the mild shortage in folate availability of nutritional folate deprivation and by comprehensive profiling of the metabolic consequences of this mild folate deprivation in erythroid cells, we identified premature, aberrant differentiation process that can explain the manifestation of megaloblastic anemia, including low erythroid cell counts and large, heme-filled cells. Therefore, our work identifies a molecular etiology for nutritional megaloblastic anemia and demonstrates that a metabolic stress can alter differentiation dynamics in a clinically relevant developmental biology model system.

## MATERIALS AND METHODS

### Cell lines

All cell lines were tested monthly and found to be negative for mycoplasma infection. The sources of the cell lines are as follows: K562, HEL, RAMOS, P12-Ichikawa, MOLM13, NALM6, NOMO, and EOL-1: D. M. Sabatini, Massachusetts Institute of Technology (MIT); MEL: M. D. Fleming, Boston Children's Hospital (BCH). All cells were cultured at 37°C with 5% CO_2_.

### Cell culture experiments

Cell lines were maintained in folate-free RPMI (Sigma-Aldrich, R1145) supplemented with 10% dialyzed fetal bovine serum (FBS), sodium bicarbonate (Sigma-Aldrich, S8761), l-glutamine (Sigma-Aldrich, G7513), and the indicated concentration of FA (Sigma-Aldrich, F8758). The reagents used in cell culture experiments are as follows: ferric ammonium citrate (Sigma-Aldrich, F5879), iron-bound transferrin (Sigma-Aldrich, T4132), deferoxamine (Sigma-Aldrich, D9533), inosine (Sigma-Aldrich, I4125), AICAR (Cayman Chemical, 10010241), SHIN1 (MedChemExpress, HY-112066), rapamycin (EMD Millipore, 553210), GSK621 (Sigma-Aldrich, SML2003), GFX (MedChemExpress, HY-13867), PMA (Cayman Chemical, 10008014), choline (Sigma-Aldrich, C7527), [2-^13^C]serine (Cambridge Isotope Laboratories, CLM-2013-PK), and [2-^13^C]glycine (Cambridge Isotope Laboratories, CLM-136-PK). The following amino acid supplements are from Sigma-Aldrich: glycine, G8790; l-arginine, A8094; l-asparagine, A4159; l-aspartic acid, A7219; l-cystine 2HCl, C6727; l-glutamic acid, G8415; l-glutamine, G8540; l-histidine, H6034; l-hydroxyproline, H5534; l-isoleucine, I7403; l-leucine, L8912; l-lysine hydrochloride, L8662; l-methionine, M5308; l-phenylalanine, P5482; l-proline, P5607; l-serine, S4311; l-threonine, T8441; l-tryptophan, T8941; l-tyrosine disodium salt dihydrate, T1145; l-valine, V0513; dimethyl sulfoxide, D2650.

All cell culture experiments were seeded with a starting cell density of 100,000 cells/ml and cultured for 2 days in the specified culture medium. After 2 days, cells were counted and reseeded at 100,000 cells/ml in the specified condition with fresh medium. Cell proliferation studies continued for 6 days, and cell doubling calculations were calculated using the equation

$${\text{Cell Doublings}}_{\text{Day}\;2}={\text{log}}_{2}\left({\text{Density}}_{\text{Day}\;2}\right)-{\text{log}}_{2}\left({\text{Density}}_{\text{Day}\;0}\right)$$

Cumulative doublings were calculated by summing the cell doublings between day 0 and day 6.

### Ex vivo erythroid progenitor expansion

Animal procedures were approved by the MIT Institutional Animal Care and Use Committee. Isolation and purification of lineage depletion and erythroid progenitor cells from fetal livers of E14.5 mice were performed as previously described (*24*). Briefly, fetal livers are homogenized in PNEG buffer [phosphate-buffered saline (PBS), 20 μM EDTA, 2% neonatal calf serum, and 10 mM glucose] and depleted of red blood cells by red blood cell lysis. Lineage depletion is achieved through magnetic pulldown with streptavidin beads and the following biotin conjugated antibodies: mouse lineage depletion panel (CD3e, CD45R, Ly-6G, Ly-6C, CD11b, and Ter119) (BD Biosciences, 559971), CD16/32, clone 93 (eBiosciences, 13-0161-82), CD41, clone eBioMWReg30 (eBiosciences, 13-0411-82), Ter119 (eBiosciences, 13-5921-82), and Ly-6A/E, clone D7 (eBiosciences, 13-5981-82). The clarified supernatant that contains CFU-E and BFU-E erythroid progenitors is retrieved, washed, and stored on ice until cell culture medium is prepared. Progenitor cells were cultured in either StemSpan SFEM II Expansion medium (STEMCELL Technologies, 09605), our custom, folate-free RPMI-based expansion medium, or progenitor differentiation medium. Both SFEM II and our custom RPMI expansion media were supplemented with SCF (100 ng/ml; PeproTech, 250-03), IGF (40 ng/ml; PeproTech, 250-19), 100 mM dexamethasone (Sigma-Aldrich, D9184), and erythropoietin (2 U/ml; PeproTech, 100-64). Our custom RPMI-based expansion medium was additionally supplemented with insulin (10 μg/ml; Sigma-Aldrich, I9278), saturated human holo-transferrin (250 μg/ml; Sigma-Aldrich, T4132), and the indicated concentration of FA. Progenitor differentiation medium was formulated by supplementing Iscove's modified Dulbecco's medium (Thermo Fisher Scientific, 12440053) with 15% FBS, saturated human holo-transferrin (250 μg/ml; Sigma-Aldrich, T4132), 2 mM l-glutamine (Thermo Fisher Scientific, 25030081), erythropoietin (1 U/ml; PeproTech, 100-64), insulin (10 μg/ml; Sigma-Aldrich, I9278), and penicillin/streptomycin. Progenitor cells were seeded under the respective medium conditions at a density of 50,000 cells/ml. Cell proliferation was assessed by cell counts at day 2 and day 4 of expansion in the indicated medium.

### Metabolite profiling by mass spectrometry

#### Polar metabolite detection

One million cells from culture were collected via centrifugation, washed with 0.9% NaCl, and resuspended in extraction buffer [80% methanol, 25 mM ammonium acetate and 2.5 mM Na-ascorbate prepared in liquid chromatography–mass spectrometry (LC-MS) water, supplemented with isotopically labeled amino acid standards (Cambridge Isotope Laboratories, MSK-A2-1.2), aminopterin, and reduced glutathione standard (Cambridge Isotope Laboratories, CNLM-6245-10)]. Samples were vortexed for 10 s and then centrifuged for 10 min at 18,000*g* to pellet cell debris. The supernatant was divided into two tubes and dried on ice using a liquid nitrogen dryer. One tube of dried sample was saved for folates metabolite detection (see below), and the second was used for polar metabolite detection. Dried samples were resuspended in 25 μl of water, and 2 μl was injected into a ZIC-pHILIC 150 × 2.1 mm (5-μm particle size) column (EMD Millipore). operated on a Vanquish Flex ultrahigh-performance LC systems (Thermo Fisher Scientific, San Jose, CA, USA). Chromatographic separation and MS data acquisition were performed as previously described (*57*).

#### Folate metabolite detection

Dried samples were prepared for folate detection similarly to that previously described (*58*). The mass spectrometer was operated in full-scan, positive ionization mode using three narrow-range scans: 438 to 450 mass/charge ratio (*m*/*z*), 452 to 462 *m*/*z*, and 470 to 478 *m*/*z*, with the resolution set at 70,000, the AGC target at 10^6^, and the maximum injection time of 150 ms. Heated electrospray ionization (HESI) settings were as follows: sheath gas flow rate, 40; Auxiliary (Aux) gas flow rate, 10; sweep gas, 0; spray voltage, 2.8 (negative) and 3.5 (positive); capillary temperature, 300; S-lens radio frequency (RF) level, 50; Aux gas heater temp, 350. Levels of folates were normalized to aminopterin as an internal standard and to polar metabolites.

#### Porphyrin metabolite detection

Porphyrin extraction was based on published protocol (*58*), with modifications. Briefly, 1 million cells from culture were collected via centrifugation, washed with 0.9% NaCl, and resuspended in 150 μl of porphyrin extraction buffer [1:4 ratio of 1.7 M HCl:acetonitrile (ACN), 1 μM deuteroporphyrin IX (Frontier Scientific, D510-9), and 0.5 μM isotopically labeled amino acids (Cambridge Isotopes, MSK-A2-1.2)]. Samples were vigorously shaken for 20 min at 16°C in a thermomixer (Eppendorf), sonicated for 10 cycles at 4°C with 30-s on and 30-s off, and then incubated at 4°C for 10 min. Following incubation on ice, samples were centrifuged for 10 min at 18,000*g* to pellet cell debris. The supernatant was collected, and 40.5 μl of supersaturated MgSO_4_ and 12 μl of 5 M NaCl were added. Samples were vortexed for 30 s and further shaken for 10 min at 16°C in a thermomixer. Last, a 10-min 10,000-rpm centrifugation was used to separate the organic layer (upper) from the aqueous layer (lower). The upper organic layer was collected, and 5 μl was injected onto a 2.6-μm, 150 × 3 mm C18 column (Phenomenex, 00F-4462-Y0) equipped with a 3.0-mm safe-guard column (Phenomenex, AJ0-8775). Column compartment was heated to 45°C. Porphyrins were separated with a chromatographic gradient at a flow rate of 0.800 ml/min as follows: 0 to 2 min, 5% B; 2 to 19 min, linear gradient from 5 to 95% B; 19 to 21 min, 95% B; 21.1 to 23 min, return to 5% B. The mass spectrometer was operated in full-scan, positive ionization mode using a narrow-range scan: 450 to 700 *m*/*z*, with an additional target selective ion monitoring (tSIM) scan for hemin (616.1767 *m*/*z*), CoproP (655.2762 *m*/*z*), and PPIX (563.2653 *m*/*z*) with the resolution set at 70,000, the AGC target at 1 × 10^6^, and the maximum injection time of 50 ms. HESI settings were as follows: sheath gas flow rate, 40; Aux gas flow rate, 10; sweep gas, 0; spray voltage, 2.8 (negative) and 3.5 (positive); capillary temperature, 300; S-lens RF level, 55; Aux gas heater temperature, 350.

### Metabolomics data analysis

Polar metabolites, folates, and porphyrins were relatively quantified while referencing an in-house library of chemical standards and using TraceFinder 4.1 (Thermo Fisher Scientific, Waltham, MA, USA), with a 5–part per million mass tolerance. Pooled samples and fractional dilutions were prepared as quality controls and injected at the beginning and end of each run. Pooled samples were interspersed throughout the run to control for technical drift in signal quality and for coefficient of variability (CV) determination for each metabolite. Data normalizations were performed in two steps: (i) Integrated peak area signal from internal standards added to extraction buffers were mean-centered (for every standard, peak area was divided by the mean peak area of the set) and averaged across samples; samples were divided by the resulting factor, thus normalizing for any technical variability due to MS signal fluctuation or pipetting and sample injection errors (usually within 10% variability). (ii) Normalization for biological material was based on detected polar metabolites as follows: CV values (based on pooled sample reinjections) and coefficient of determination (R-squared) (based on linear dilutions of pooled sample) were calculated per metabolite, metabolites with <30% CV and >0.95 RSQ were mean-centered and averaged across samples. Metabolite peak areas were then divided by the resulting factor (biological normalizer), thus accounting for any global shift in metabolite amounts due to differences in biological material.

### Serine and glycine tracing

Tracing experiments were conducted in folate, B12, and amino acid–free RPMI (US Bio, R9010-06) supplemented with 10% dialyzed FBS, B12 (Sigma-Aldrich, V6629), and amino acids (to the standard RPMI concentration, see below for product information). Before tracing, cells were cultured as described above in amino acid–free RPMI supplemented with all unlabeled amino acids. [2-^13^C]serine and [2-^13^C]glycine tracing was performed in K562 cells following 8 days culture in 2000 nM or 100 nM FA. At day 8, cells were washed and placed in amino acid–free RPMI at the indicated FA concentration containing all amino acids minus serine and glycine. As indicated, cells were supplemented with [unlabeled]serine + [unlabeled]glycine, [2-^13^C]serine + [unlabeled]glycine, or [unlabeled]serine + [2-^13^C]glycine. Total glycine and serine concentrations under all medium conditions were 10 and 30 mg/liter, respectively. Following 24 hours of culture under labeled or unlabeled conditions, cells were quickly washed in 0.9% NaCl and extracted in either polar or porphyrin extraction buffer. Relative quantification of polar metabolites was performed with TraceFinder 4.1 as described above. To identify metabolites with expected ^13^C labeling, the mass of the extra proton (*m* = 1.00727) was added to the expected *m*/*z* for each potential carbon that could be labeled. Relative quantification of this list of compounds allowed the calculation of percent labeling for each carbon (*m* + 1, *m* + 2, *m* + 3, etc.). Subtraction of the natural abundance of ^13^C was performed using the R package, IsoCorrectoR (*59*). Corrected abundances were presented as the change in percent labeling between experimental conditions.

### Immunoblot

Cell lysis was performed with RIPA buffer (Santa Cruz Biotechnology, sc-24948) following the manufacturer’s protocol. Protein concentration was measured using bicinchoninic acid assay (Thermo Fisher Scientific, 23227). Protein samples were prepared in 2× sample buffer (Thermo Fisher Scientific, LC2676) so that 20 μg of protein lysate per sample could be used for SDS–polyacrylamide gel electrophoresis. Immunoblotting was performed using the following primary antibodies: hemoglobin A1 (Abcam, ab92492; 1:1000), hemoglobin, fetal (Abcam, ab137096; 1:1000), p-ULK1-S555 [Cell Signaling Technology (CST), 5869T; 1:1000], ULK1 (CST, 8054S; 1:1000), p-ACC-S79 (CST, 3661P; 1:1000), ACC (CST, 3676P; 1:1000), vinculin (CST, 13901; 1:2500), p-S6-T389 (CST, 9205; 1:1000), S6 (CST, 9202; 1:1000), AMPKa (CST, 5832; 1:1000), SHMT2 (CST, 33443; 1:1000), caspase-3 (CST, 14220; 1:1000), cleaved caspase-3 (CST, 9661; 1:1000); PKC-δ (CST, 9616; 1:1000), PKC-ζ (CST, 9368; 1:1000), phospho-PKC-ζ/λ (CST, 9378; 1:1000), PKD/PKC-μ (CST, 90039; 1:1000), phospho-PKC-θ (CST, 9377; 1:1000), phospho-PKC-δ (CST, 9374; 1:1000), phospho-PKC-α/β II (CST, 9375; 1:1000), phospho-PKC (CST, 9371; 1:1000), PKC-α (Abcam, ab180848; 1:1000), and phospho-(Ser) PKC (CST, 2261; 1:1000). Horseradish peroxidase–conjugated anti-rabbit (Jackson ImmunoResearch, 111-035-144; 1:10,000) and anti-mouse (Jackson ImmunoResearch, 115-035-166; 1:10,000) secondary antibodies were used.

### Flow cytometry

Flow cytometry was performed on a BD FACSCelesta. Briefly, cells were washed with fluorescence-activated cell sorting (FACS) buffer (PBS + 2% FBS) and incubated for 20 min in the presence of the indicated antibodies. Cells were washed in FACS buffer, and viability was assessed using 4′,6-diamidino-2-phenylindole (DAPI) (1 μg/ml; Sigma-Aldrich, D9542). The following flow cytometry antibodies were used: Ter119 (BioLegend, 116206; 1:200), GlyA (BioLegend, 349104; 1:200), transferrin receptor (mouse) (BioLegend, 113819; 1:200), and transferrin receptor (human) (BioLegend, 334108; 1:200).

### Benzidine staining

Benzidine staining was performed using a stock of 0.2% tetremethylbenzidine (TMB) (Sigma-Aldirch, T8768) in 0.5% acetic acid (Fisher Chemical, BP1185-500) and deionized water. Just before use, 30% hydrogen peroxide was added to the stock solution at a concentration of 0.5% to generate a working solution. Cells were washed once with PBS and resuspended in 0.20 ml of PBS. A total of 0.20 ml of the 0.2% TMB working solution was added to the cells and incubated for 10 min at room temperature in the dark. The cells were imaged using a bright-field imaging microscope. Bright-field imaging was accomplished using the Nikon Eclipse Ts2 inverted phase contrast microscope using a Nikon Digital Sight 1000 camera and 10× and 20× objectives. Staining was quantified by cell counting.

### Immunofluorescence assay

Approximately 2 × 10^5^ K562 and MEL cells in PBS were centrifuged (300 rpm for 5 min) onto glass slides using a Cytospin 4 cytocentrifuge (Thermo Fisher Scientific). Cells were fixed for 15 min with 4% paraformaldehyde and permeabilized with 0.1% Triton X-100 in PBS for 10 min. Cells were then blocked with 10% goat serum (Colorado Serum Company, 30920) for 1 hour at room temperature and incubated with phospho-histone H2A.X (Ser^139^) antibody (CST, #2577) in blocking solution overnight at 4°C. Cells were washed three times with PBS, followed by incubation with Alexa Fluor 488–conjugated secondary antibody (Abcam, ab150077) for an hour at room temperature. Cells were then stained with DAPI (0.1 μg/ml; Sigma-Aldrich, D9542) for 5 min and washed three times with PBS. Coverslips were mounted onto glass slides using ProLong Gold Antifade Mountant (Thermo Fisher Scientific, P36930). Images were acquired using Abberior STEDYCON microscope. Images were analyzed with CellProfiler (*60*). Phospho-histone H2A.X fluorescence intensity was measured for each nucleus and normalized to nuclear area.

### Gene expression analysis (RNA, cDNA, and quantitative polymerase chain reaction)

RNA extraction from cell pellets was performed using RNAzol (Thermo Fisher Scientific, NC0477546). The reverse transcriptase reaction was performed using between 100 ng and 1 μg of RNA per sample (New England Biolabs, E3010). Quantitative polymerase chain reaction (qPCR) reactions were performed with SYBR green (CWBiotech, CW2621) on the QuantStudio 7 Pro (Applied Biosystems) qPCR instrument. The reference gene for all qPCR reactions were β-actin.

Primers used for qPCR are the following: hActin, CAACCGCGAGAAGATGACCC (forward) and AGGCGTACAGGGATAGCACA (reverse); mActin, GGCTGTATTCCCCTCCATCG (forward) and CCAGTTGGTAACAATGCCATGT (reverse); hALAS2, CACCACAGCCCTCAGATGAT (forward) and CAAAGTGTACAGGACGGCGA (reverse); mALAS2, GGGCTAAGAGCCATTGTCCT (forward) and ATGGCTTCGGGTGGTTGAAT (reverse); hALAS1, AGATCTGACCCCTCAGTCCC (forward) and TCCACGAAGGTGATTGCTCC (reverse); ALAD, GGCTACTTCCACCCACTACTT (forward) and ATGGAAGTGGAGATGGTGGGA (reverse); HMBS, GCAGGAGTTCAGTGCCATCATC (forward) and AGCACACCCACCAGATCCAAG (reverse); UROS, CAGTTGCACACCCAGGAATC (forward) and ATGTGAGGCCAGAGGGACTAA (reverse); UROD, GCTGACCATGGAAGCGAATG (forward) and TGCACCAAACGGGAGTGTAG (reverse); CPOX, TGGGCGTGAGCTCTGTTATC (forward) and CACCAAACCACCACTGCTTG (reverse); PPOX, GAACAGGTTCCTCTACGTGGG (forward) and GAGACGCCACCTCAGGTCC (reverse); FECH, AAAGGGCTTTGTGAGAGGGG (forward) and CAGGGCCTTAGAGAACAATGGA (reverse); MTHFS, GAGTGATTGCCCACAGTGAGT (forward) and TTTGCCTCGTTGGAAAATGT (reverse); SHMT1, GAGCAGTTTTGGAGGCCCTA (forward) and CTCGCTTCTGACAGAGGGTC (reverse); SHMT2, CCTAGACCAGAGTTGGTGGC (forward) and TGTTTGCTTCCCCAGTCTGA (reverse); MTHFD1, GTGCACTCACTGGGCAGAAG (forward) and TCAGCTTTGTGTTGAGCTTCG (reverse); MTHFD2, GGTCTATGGCTGCGACTTCTCT (forward) and TTGATCTGCTGGGCCAGTTTC (reverse); SLC19A1, GAGAGCTTCATCACCCCCTA (forward) and TGTGCATCTCCCAAAGTGTG (reverse); TYMS, GGTGTGCCTTTCAACATC (forward) and GGCCAGGTAGGAGTACGACA (reverse).

### Mitochondrial analysis

Mitochondrial mass was assessed using the mitochondrial permeable, potential independent dye Mitospy (BioLegend, 424805). Mitochondrial potential was assessed using the potential dependent dye, Mitotracker deep red (Invitrogen, M22426). Cells were incubated at 37°C for 30 min in the presence of dye and then prepared for flow cytometry as described above. The Seahorse XFe96 Analyzer was used to assess oxygen consumption rate (OCR) in K562. Before OCR measurement, cells were washed and placed in a custom, folate-free RPMI medium lacking glutamine, pyruvate, glucose, phenol red, and sodium bicarbonate (Gibco). The medium was supplemented with pyruvate, glucose, and l-glutamine immediately before experiment. K562 were adhered to the 96-well plate using Cell-Tak and then incubated at 37°C for 1 hour in an incubator without CO_2_. The standard mitochondrial stress test was performed with injection of carbonyl cyanide *p*-trifluoromethoxyphenylhydrazone (2 μM; Sigma-Aldrich, C2920), oligomycin (1 μM; Sigma-Aldrich, 75351), rotenone (500 nM; Sigma-Aldrich, R8875), and antimycin A (500 nM; Sigma-Aldrich, A8674). Analysis was performed using Wave software (Agilent Technologies).

### RNA sequencing

RNA was isolated using RNAzol, following the manufacturer’s protocols (Thermo Fisher Scientific, NC0477546). Bulk RNA was sequenced utilizing GeneWiz’s standard RNA-seq platform. We received Fastq files and performed analysis using the Boston Children’s Hospital E2 Computer Cluster. Briefly, quality control was performed via fastQC (www.bioinformatics.babraham.ac.uk/projects/fastqc/). Reads were mapped to the mouse or human transcriptome using STAR aligner (*61*). Transcript quantification was performed using RSEM (*62*). Transcript abundance was imported into R using tximport, and differential gene expression analysis was performed using DeSeq2 (*63*, *64*). GSEA was done using the Broad Institute’s GSEA software (GSEA v4.3.2) (*65*). The MSigDB Hallmark gene set was used for all GSEA analysis.

### Statistical analysis and software

Analysis and visualization of metabolomics data were generated with MetaboAnalyst 5.0 (www.metaboanalyst.ca). Statistical analysis was performed using GraphPad Prism 9. For pairwise comparisons, two-tailed Student’s *t* tests were used. For multiple comparisons, one-way analysis of variance (ANOVA) test with Tukey’s post hoc test was used. All error bars represent SD.

## References

[1] G. H. Hitchings, J. J. Burchall, Inhibition of folate biosynthesis and function as a basis for chemotherapy. Adv. Enzymol. Relat. Areas Mol. Biol. 27, 417–468 (1965). 4387360 10.1002/9780470122723.ch9

[2] K. S. Crider, T. P. Yang, R. J. Berry, L. B. Bailey, Folate and DNA methylation: A review of molecular mechanisms and the evidence for folate’s role. Adv. Nutr. 3, 21–38 (2012). 22332098 10.3945/an.111.000992 PMC3262611

[3] M. C. De Santis, P. E. Porporato, M. Martini, A. Morandi, Signaling pathways regulating redox balance in cancer metabolism. Front. Oncol. 8, 126 (2018). 29740540 10.3389/fonc.2018.00126 PMC5925761

[4] G. S. Ducker, J. D. Rabinowitz, One-carbon metabolism in health and disease. Cell Metab. 25, 27–42 (2017). 27641100 10.1016/j.cmet.2016.08.009 PMC5353360

[5] J. F. Wilkinson, M. C. Israels, F. Fletcher, Folic acid in the treatment of pernicious anaemia. Lancet 2, 156–158 (1946). 20995252

[6] L. C. Dore, J. D. Crispino, Transcription factor networks in erythroid cell and megakaryocyte development. Blood 118, 231–239 (2011). 21622645 10.1182/blood-2011-04-285981 PMC3138678

[7] D. W. Smith, The molecular biology of mammalian hemoglobin synthesis. Ann. Clin. Lab. Sci. 10, 116–122 (1980). 6992694

[8] M. J. Koury, D. W. Horne, Apoptosis mediates and thymidine prevents erythroblast destruction in folate deficiency anemia. Proc. Natl. Acad. Sci. U.S.A. 91, 4067–4071 (1994). 8171036 10.1073/pnas.91.9.4067 PMC43724

[9] M. J. Koury, D. W. Horne, Z. A. Brown, J. A. Pietenpol, B. C. Blount, B. N. Ames, R. Hard, S. T. Koury, Apoptosis of late-stage erythroblasts in megaloblastic anemia: Association with DNA damage and macrocyte production. Blood 89, 4617–4623 (1997). 9192787

[10] G. Robert, I. Ben Sahra, A. Puissant, P. Colosetti, N. Belhacene, P. Gounon, P. Hofman, F. Bost, J. P. Cassuto, P. Auberger, Acadesine kills chronic myelogenous leukemia (CML) cells through PKC-dependent induction of autophagic cell death. PLOS ONE 4, e7889 (2009). 19924252 10.1371/journal.pone.0007889 PMC2775681

[11] N. L. Lumelsky, B. S. Schwartz, Protein kinase C in erythroid and megakaryocytic differentiation: Possible role in lineage determination. Biochim. Biophys. Acta 1358, 79–92 (1997). 9296525 10.1016/s0167-4889(97)00051-7

[12] J. H. Myklebust, E. B. Smeland, D. Josefsen, M. Sioud, Protein kinase C-α isoform is involved in erythropoietin-induced erythroid differentiation of CD34^+^ progenitor cells from human bone marrow. Blood 95, 510–518 (2000). 10627456

[13] J. A. Rivero, S. E. Adunyah, Sodium butyrate stimulates PKC activation and induces differential expression of certain PKC isoforms during erythroid differentiation. Biochem. Biophys. Res. Commun. 248, 664–668 (1998). 9703983 10.1006/bbrc.1998.9041

[14] J. R. Cantor, M. Abu-Remaileh, N. Kanarek, E. Freinkman, X. Gao, A. Louissaint Jr., C. A. Lewis, D. M. Sabatini, Physiologic medium rewires cellular metabolism and reveals uric acid as an endogenous inhibitor of ump synthase. Cell 169, 258–272.e17 (2017). 28388410 10.1016/j.cell.2017.03.023 PMC5421364

[15] D. L. McKay, G. Perrone, H. Rasmussen, G. Dallal, J. B. Blumberg, Multivitamin/mineral supplementation improves plasma B-vitamin status and homocysteine concentration in healthy older adults consuming a folate-fortified diet. J. Nutr. 130, 3090–3096 (2000). 11110875 10.1093/jn/130.12.3090

[16] L. M. Rogers, A. M. Cordero, C. M. Pfeiffer, D. B. Hausman, B. L. Tsang, L. M. de-Regil, J. Rosenthal, H. Razzaghi, E. C. Wong, A. P. Weakland, L. B. Bailey, Global folate status in women of reproductive age: A systematic review with emphasis on methodological issues. Ann. N. Y. Acad. Sci. 1431, 35–57 (2018). 30239016 10.1111/nyas.13963 PMC6282622

[17] L. C. Andersson, K. Nilsson, C. G. Gahmberg, K562—A human erythroleukemic cell line. Int. J. Cancer 23, 143–147 (1979). 367973 10.1002/ijc.2910230202

[18] C. B. Lozzio, B. B. Lozzio, Human chronic myelogenous leukemia cell-line with positive Philadelphia chromosome. Blood 45, 321–334 (1975). 163658

[19] J. E. Baggott, T. Tamura, Folate-dependent purine nucleotide biosynthesis in humans. Adv. Nutr. 6, 564–571 (2015). 26374178 10.3945/an.115.008300 PMC4561830

[20] A. W. Murray, The biological significance of purine salvage. Annu. Rev. Biochem. 40, 811–826 (1971). 4330582 10.1146/annurev.bi.40.070171.004115

[21] P. Ponka, Cell biology of heme. Am. J. Med. Sci. 318, 241–256 (1999). 10522552 10.1097/00000441-199910000-00004

[22] H. A. Dailey, P. N. Meissner, Erythroid heme biosynthesis and its disorders. Cold Spring Harb. Perspect. Med. 3, a011676 (2013). 23471474 10.1101/cshperspect.a011676 PMC3683999

[23] D. Singer, M. Cooper, G. M. Maniatis, P. A. Marks, R. A. Rifkind, Erythropoietic differentiation in colonies of cells transformed by Friend virus. Proc. Natl. Acad. Sci. U.S.A. 71, 2668–2670 (1974). 4527790 10.1073/pnas.71.7.2668 PMC388528

[24] P. Martin, T. Papayannopoulou, HEL cells: A new human erythroleukemia cell line with spontaneous and induced globin expression. Science 216, 1233–1235 (1982). 6177045 10.1126/science.6177045

[25] V. G. Sankaran, T. F. Menne, J. Xu, T. E. Akie, G. Lettre, B. van Handel, H. K. A. Mikkola, J. N. Hirschhorn, A. B. Cantor, S. H. Orkin, Human fetal hemoglobin expression is regulated by the developmental stage-specific repressor BCL11A. Science 322, 1839–1842 (2008). 19056937 10.1126/science.1165409

[26] M. J. Koury, J. O. Price, G. G. Hicks, Apoptosis in megaloblastic anemia occurs during DNA synthesis by a p53-independent, nucleoside-reversible mechanism. Blood 96, 3249–3255 (2000). 11050010

[27] N. Lamm, K. Maoz, A. C. Bester, M. M. Im, D. S. Shewach, R. Karni, B. Kerem, Folate levels modulate oncogene-induced replication stress and tumorigenicity. EMBO Mol. Med. 7, 1138–1152 (2015). 26197802 10.15252/emmm.201404824 PMC4568948

[28] B. T. Do, P. P. Hsu, S. Y. Vermeulen, Z. Wang, T. Hirz, K. L. Abbott, N. Aziz, J. M. Replogle, S. Bjelosevic, J. Paolino, S. Nelson, S. Block, A. M. Darnell, R. Ferreira, H. Zhang, J. Milosevic, D. R. Schmidt, C. Chidley, I. S. Harris, J. S. Weissman, Y. Pikman, K. Stegmaier, S. Cheloufi, X. A. Su, D. B. Sykes, M. G. Vander Heiden, Nucleotide depletion promotes cell fate transitions by inducing DNA replication stress. bioRxiv (2022) doi: 10.1101/2022.08.16.503984.

[29] F. Osti, F. G. Corradini, S. Hanau, M. Matteuzzi, R. Gambari, Human leukemia K562 cells: Induction to erythroid differentiation by guanine, guanosine and guanine nucleotides. Haematologica 82, 395–401 (1997). 9299849

[30] J. F. Gusella, D. Housman, Induction of erythroid differentiation in vitro by purines and purine analogues. Cell 8, 263–269 (1976). 971485 10.1016/0092-8674(76)90010-6

[31] E. Olah, Y. Natsumeda, T. Ikegami, Z. Kote, M. Horanyi, J. Szelenyi, E. Paulik, T. Kremmer, S. R. Hollan, J. Sugar, Induction of erythroid differentiation and modulation of gene expression by tiazofurin in K-562 leukemia cells. Proc. Natl. Acad. Sci. U.S.A. 85, 6533–6537 (1988). 2901100 10.1073/pnas.85.17.6533 PMC282007

[32] M. Ichii, K. Oritani, M. Murase, K. Komatsu, M. Yamazaki, R. Kyoden, N. Kito, Y. Nozaki, M. Saito, H. Iwamura, Y. Kanakura, Molecular targeting of inosine-5′-monophosphate dehydrogenase by FF-10501 promotes erythropoiesis via ROS/MAPK pathway. Leuk. Lymphoma 59, 448–459 (2018). 28730859 10.1080/10428194.2017.1339878

[33] G. S. Ducker, J. M. Ghergurovich, N. Mainolfi, V. Suri, S. K. Jeong, S. Hsin-Jung Li, A. Friedman, M. G. Manfredi, Z. Gitai, H. Kim, J. D. Rabinowitz, Human SHMT inhibitors reveal defective glycine import as a targetable metabolic vulnerability of diffuse large B-cell lymphoma. Proc. Natl. Acad. Sci. U.S.A. 114, 11404–11409 (2017). 29073064 10.1073/pnas.1706617114 PMC5664509

[34] J. Chung, C. Chen, B. H. Paw, Heme metabolism and erythropoiesis. Curr. Opin. Hematol. 19, 156–162 (2012). 22406824 10.1097/MOH.0b013e328351c48b PMC4086261

[35] J. Zhang, H. F. Lodish, Endogenous K-ras signaling in erythroid differentiation. Cell Cycle 6, 1970–1973 (2007). 17721087 10.4161/cc.6.16.4577

[36] J. Zhang, M. Socolovsky, A. W. Gross, H. F. Lodish, Role of Ras signaling in erythroid differentiation of mouse fetal liver cells: Functional analysis by a flow cytometry-based novel culture system. Blood 102, 3938–3946 (2003). 12907435 10.1182/blood-2003-05-1479

[37] G. Hoxhaj, J. Hughes-Hallett, R. C. Timson, E. Ilagan, M. Yuan, J. M. Asara, I. Ben-Sahra, B. D. Manning, The mTORC1 signaling network senses changes in cellular purine nucleotide levels. Cell Rep. 21, 1331–1346 (2017). 29091770 10.1016/j.celrep.2017.10.029 PMC5689476

[38] J. M. Corton, J. G. Gillespie, S. A. Hawley, D. G. Hardie, 5-aminoimidazole-4-carboxamide ribonucleoside. A specific method for activating AMP-activated protein kinase in intact cells? Eur. J. Biochem. 229, 558–565 (1995). 7744080 10.1111/j.1432-1033.1995.tb20498.x

[39] M. M. Mihaylova, R. J. Shaw, The AMPK signalling pathway coordinates cell growth, autophagy and metabolism. Nat. Cell Biol. 13, 1016–1023 (2011). 21892142 10.1038/ncb2329 PMC3249400

[40] P. Sujobert, L. Poulain, E. Paubelle, F. Zylbersztejn, A. Grenier, M. Lambert, E. C. Townsend, J. M. Brusq, E. Nicodeme, J. Decrooqc, I. Nepstad, A. S. Green, J. Mondesir, M. A. Hospital, N. Jacque, A. Christodoulou, T. A. Desouza, O. Hermine, M. Foretz, B. Viollet, C. Lacombe, P. Mayeux, D. M. Weinstock, I. C. Moura, D. Bouscary, J. Tamburini, Co-activation of AMPK and mTORC1 induces cytotoxicity in acute myeloid leukemia. Cell Rep. 11, 1446–1457 (2015). 26004183 10.1016/j.celrep.2015.04.063

[41] H. C. Chen, G. Bandyopadhyay, M. P. Sajan, Y. Kanoh, M. Standaert, R. V. Farese Jr., R. V. Farese, Activation of the ERK pathway and atypical protein kinase C isoforms in exercise- and aminoimidazole-4-carboxamide-1-β-d-riboside (AICAR)-stimulated glucose transport. J. Biol. Chem. 277, 23554–23562 (2002). 11978788 10.1074/jbc.M201152200

[42] N. J. Huang, Y. C. Lin, C. Y. Lin, N. Pishesha, C. A. Lewis, E. Freinkman, C. Farquharson, J. L. Millán, H. Lodish, Enhanced phosphocholine metabolism is essential for terminal erythropoiesis. Blood 131, 2955–2966 (2018). 29712634 10.1182/blood-2018-03-838516 PMC6024642

[43] T. W. Braun, M. K. Kuoch, E. Khandros, H. Li, FACS and immunomagnetic isolation of early erythroid progenitor cells from mouse fetal liver. STAR Protoc 3, 101070 (2022). 35024628 10.1016/j.xpro.2021.101070 PMC8724924

[44] H. Li, J. Shi, N. J. Huang, N. Pishesha, A. Natarajan, J. C. Eng, H. F. Lodish, Efficient CRISPR-Cas9 mediated gene disruption in primary erythroid progenitor cells. Haematologica 101, e216–e219 (2016). 26969085 10.3324/haematol.2015.135723 PMC5013953

[45] M. J. Koury, Tracking erythroid progenitor cells in times of need and times of plenty. Exp. Hematol. 44, 653–663 (2016). 26646992 10.1016/j.exphem.2015.10.007

[46] C. Giles, E. M. Shuttleworth, Megaloblastic anaemia of pregnancy and the puerperium. Lancet 2, 1341–1347 (1958). 13612218 10.1016/s0140-6736(58)91437-5

[47] C. C. Ungley, Folic acid, vitamin B12 and anemia. III. Folic acid and vitamin B12 in megaloblastic anaemia. J. Pharm. Pharmacol. 2, 540–544 (1950). 14779275 10.1111/j.2042-7158.1950.tb12971.x

[48] J. Chandra, Megaloblastic anemia: Back in focus. Indian J. Pediatr. 77, 795–799 (2010). 20589460 10.1007/s12098-010-0121-2

[49] C. F. Ingram, A. F. Fleming, M. Patel, J. S. Galpin, Pregnancy- and lactation-related folate deficiency in South Africa--A case for folate food fortification. S. Afr. Med. J. 89, 1279–1284 (1999). 10678198

[50] C. M. Pfeiffer, M. R. Sternberg, M. Zhang, Z. Fazili, R. J. Storandt, K. S. Crider, S. Yamini, J. J. Gahche, W. Y. Juan, C. Y. Wang, N. Potischman, J. Williams, D. J. LaVoie, Folate status in the US population 20 y after the introduction of folic acid fortification. Am. J. Clin. Nutr. 110, 1088–1097 (2019). 31504109 10.1093/ajcn/nqz184 PMC6821545

[51] M. J. Koury, P. Ponka, New insights into erythropoiesis: The roles of folate, vitamin B12, and iron. Annu. Rev. Nutr. 24, 105–131 (2004). 15189115 10.1146/annurev.nutr.24.012003.132306

[52] L. W. Sullivan, Differential diagnosis and management of the patient with megaloblastic anemia. Am. J. Med. 48, 609–617 (1970). 4912934 10.1016/0002-9343(70)90011-2

[53] A. N. Bond, G. Harris, S. N. Wickramasinghe, DNA chain elongation rates in marrow cells from vitamin B12-deficient patients and methotrexate-treated mice. Br. J. Haematol. 50, 299–307 (1982). 6277361 10.1111/j.1365-2141.1982.tb01920.x

[54] S. N. Wickramasinghe, The wide spectrum and unresolved issues of megaloblastic anemia. Semin. Hematol. 36, 3–18 (1999). 9930565

[55] Y. Hong, J. F. Martin, W. Vainchenker, J. D. Erusalimsky, Inhibition of protein kinase C suppresses megakaryocytic differentiation and stimulates erythroid differentiation in HEL cells. Blood 87, 123–131 (1996). 8547633

[56] M. von Lindern, M. P. V. Amelsvoort, T. van Dijk, E. Deiner, E. van den Akker, S. van Emst-de Vries, P. Willems, H. Beug, B. Löwenberg, Protein kinase C alpha controls erythropoietin receptor signaling. J. Biol. Chem. 275, 34719–34727 (2000). 10940312 10.1074/jbc.M007042200

[57] B. Petrova, A. Warren, N. Y. Vital, A. J. Culhane, A. G. Maynard, A. Wong, N. Kanarek, Redox metabolism measurement in mammalian cells and tissues by LC-MS. Metabolites 11, (2021). 10.3390/metabo11050313 PMC8153172 34068241

[58] J. Fyrestam, C. Östman, Determination of heme in microorganisms using HPLC-MS/MS and cobalt(III) protoporphyrin IX inhibition of heme acquisition in Escherichia coli. Anal. Bioanal. Chem. 409, 6999–7010 (2017). 29043383 10.1007/s00216-017-0610-5 PMC5717118

[59] P. Heinrich, C. Kohler, L. Ellmann, P. Kuerner, R. Spang, P. J. Oefner, K. Dettmer, Correcting for natural isotope abundance and tracer impurity in MS-, MS/MS- and high-resolution-multiple-tracer-data from stable isotope labeling experiments with IsoCorrectoR. Sci. Rep. 8, 17910 (2018). 30559398 10.1038/s41598-018-36293-4 PMC6297158

[60] D. R. Stirling, M. J. Swain-Bowden, A. M. Lucas, A. E. Carpenter, B. A. Cimini, A. Goodman, CellProfiler 4: Improvements in speed, utility and usability. BMC Bioinformatics 22, 433 (2021). 34507520 10.1186/s12859-021-04344-9 PMC8431850

[61] A. Dobin, C. A. Davis, F. Schlesinger, J. Drenkow, C. Zaleski, S. Jha, P. Batut, M. Chaisson, T. R. Gingeras, STAR: Ultrafast universal RNA-seq aligner. Bioinformatics 29, 15–21 (2013). 23104886 10.1093/bioinformatics/bts635 PMC3530905

[62] B. Li, C. N. Dewey, RSEM: Accurate transcript quantification from RNA-seq data with or without a reference genome. BMC Bioinformatics 12, 323 (2011). 21816040 10.1186/1471-2105-12-323 PMC3163565

[63] C. Soneson, M. I. Love, M. D. Robinson, Differential analyses for RNA-seq: Transcript-level estimates improve gene-level inferences. F1000Res 4, 1521 (2015). 26925227 10.12688/f1000research.7563.1 PMC4712774

[64] M. I. Love, W. Huber, S. Anders, Moderated estimation of fold change and dispersion for RNA-seq data with DESeq2. Genome Biol. 15, 550 (2014). 25516281 10.1186/s13059-014-0550-8 PMC4302049

[65] A. Subramanian, P. Tamayo, V. K. Mootha, S. Mukherjee, B. L. Ebert, M. A. Gillette, A. Paulovich, S. L. Pomeroy, T. R. Golub, E. S. Lander, J. P. Mesirov, Gene set enrichment analysis: A knowledge-based approach for interpreting genome-wide expression profiles. Proc. Natl. Acad. Sci. U.S.A. 102, 15545–15550 (2005). 16199517 10.1073/pnas.0506580102 PMC1239896

[66] R. Edgar, M. Domrachev, A. E. Lash, Gene expression omnibus: NCBI gene expression and hybridization array data repository. Nucleic Acids Res. 30, 207–210 (2002). 11752295 10.1093/nar/30.1.207 PMC99122

