# Supplementary material for: Folate depletion induces erythroid differentiation through perturbation of de novo purine synthesis
Source: Sci Adv. 2024 Jan 31;10(5):eadj9479. doi: 10.1126/sciadv.adj9479 (PMC10830111; doi:10.1126/sciadv.adj9479)
Supplement: Supplementary file 1 — Figs. S1 to S10 Legends for data S1 to S3 [file sciadv.adj9479_sm.pdf]

Science Advances
AAAS

Supplementary Materials for

Folate depletion induces erythroid differentiation through perturbation of de novo purine synthesis

Adam G. Maynard *et al.*

Corresponding author: Naama Kanarek, naama.kanarek@childrens.harvard.edu

Sci. Adv. **10**, eadj9479 (2024)
DOI: 10.1126/sciadv.adj9479

The PDF file includes:

Figs. S1 to S10
Legends for data S1 to S3

Other Supplementary Material for this manuscript includes the following:

Data S1 to S3

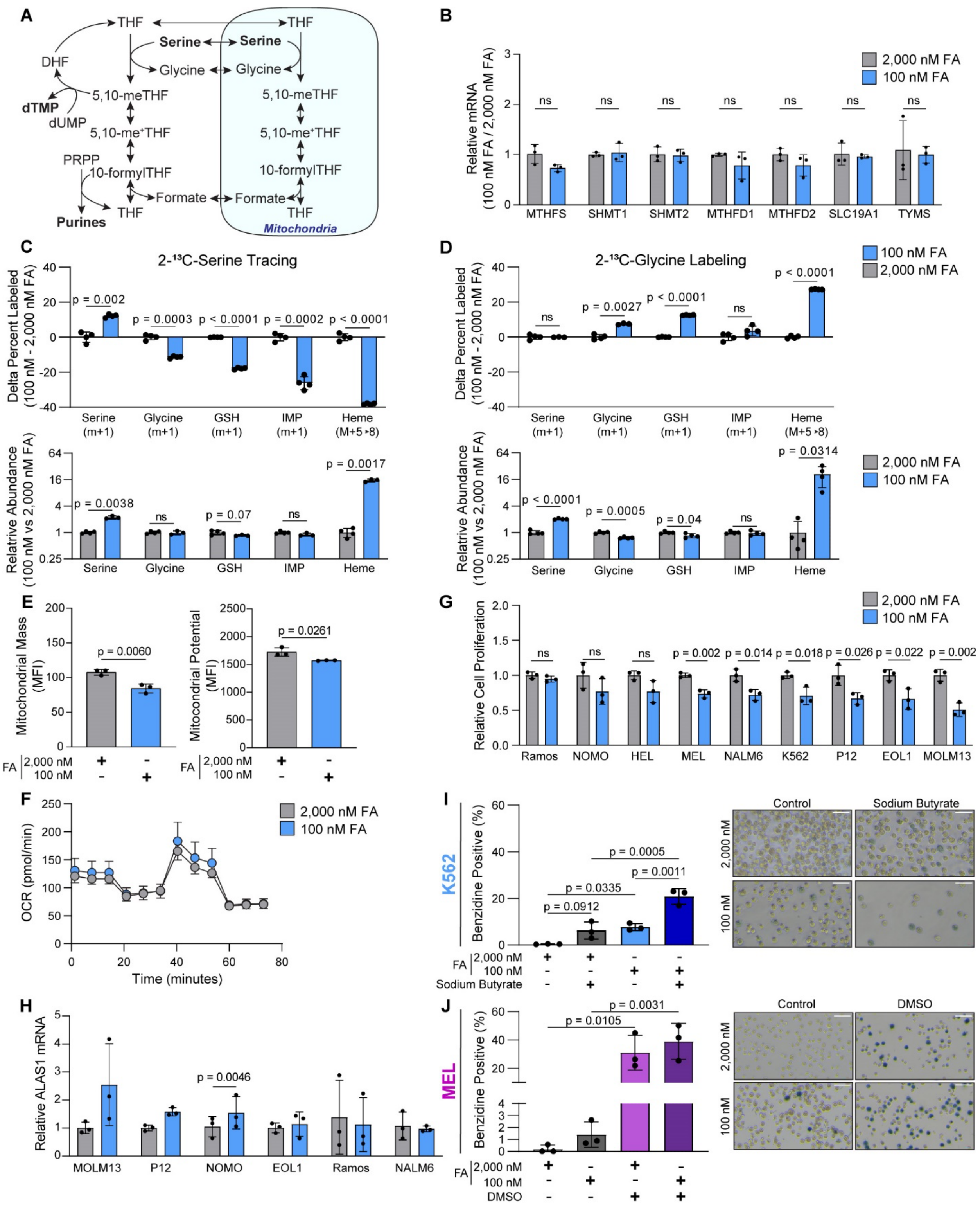

A
THF
Serine
Glycine
DHF
dTMP
dUMP
5,10-meTHF
5,10-me+THF
PRPP
10-formylTHF
Formate
Purines
Mitochondria
B
Relative mRNA (100 nM FA / 2,000 nM FA)
2,000 nM FA
100 nM FA
ns
MTHFS
SHMT1
SHMT2
MTHFD1
MTHFD2
SLC19A1
TYMS
C
2-13C-Serine Tracing
Delta Percent Labeled (100 nM - 2,000 nM FA)
p = 0.002
p = 0.0003
p < 0.0001
p = 0.0002
p < 0.0001
Serine (m+1)
Glycine (m+1)
GSH (m+1)
IMP (m+1)
Heme (M+5 ›8)
Relative Abundance (100 nM vs 2,000 nM FA)
p = 0.0038
p = 0.07
p = 0.0017
Serine
Glycine
GSH
IMP
Heme
D
2-13C-Glycine Labeling
p = 0.0027
p < 0.0001
p = 0.0314
p = 0.0005
p = 0.04
E
Mitochondrial Mass (MFI)
p = 0.0060
Mitocondrial Potential (MFI)
p = 0.0261
FA
2,000 nM
100 nM
F
OCR (pmol/min)
Time (minutes)
G
Relative Cell Proliferation
p = 0.002
p = 0.014
p = 0.018
p = 0.026
p = 0.022
Ramos
NOMO
HEL
MEL
NALM6
K562
P12
EOL1
MOLM13
H
Relative ALAS1 mRNA
p = 0.0046
I
K562
Benzidine Positive (%)
p = 0.0005
p = 0.0011
p = 0.0335
p = 0.0912
Sodium Butyrate
Control
J
MEL
p = 0.0031
p = 0.0105
DMSO

Supplementary Figure 1. Metabolic changes in erythroid cells following folate deprivation. **(A)** Simplified schematic of one-carbon metabolism. **(B)** RT-qPCR analysis of 7 genes related to 1-carbon (1C) metabolism in K562 following 6 days culture in 2,000 and 100 nM FA. **(C, D)** Difference in ^{13}C-labeling (top) or total levels (bottom) in serine, glycine, glutathione (GSH), inosine monophosphate (IMP), and Heme from (**C**) 2-^{13}C-Serine and (**D**) 2-^{13}C-Glycine following 24hr isotope labeling in K562 cells cultured in 2,000 or 100 nM FA. **(E)** Flow cytometry measurement of mitochondrial mass and membrane potential as quantified by mean fluorescence intensity (MFI) of Mitospy (mass) and Mitotracker (membrane potential). Data shown for K562 in 2,000 and 100 nM FA at day 6. **(F)** Oxygen consumption rate (OCR) from the Seahorse mitochondrial stress test in K562 at day 6 in 2,000 and 100 nM FA. **(G)** Relative proliferation rate of nine leukemia cell lines over 6 days in 2,000 and 100 nM FA. **(H)** RT-qPCR analysis of ALAS1 mRNA expression in non-erythroid cell lines. **(I)** Benzidine positive K562 following 8-day culture in 2,000 nM or 100 nM FA, in the presence or absence of sodium butyrate. Scale bar = 100 μM **(J)** Benzidine positive MEL following 8-day culture in 2,000 nM or 100 nM FA, in the presence or absence of DMSO. Scale bar = 100 μM All data shown are mean (± s.d.) of three biological replicates except: (**1C, D**) n=4.

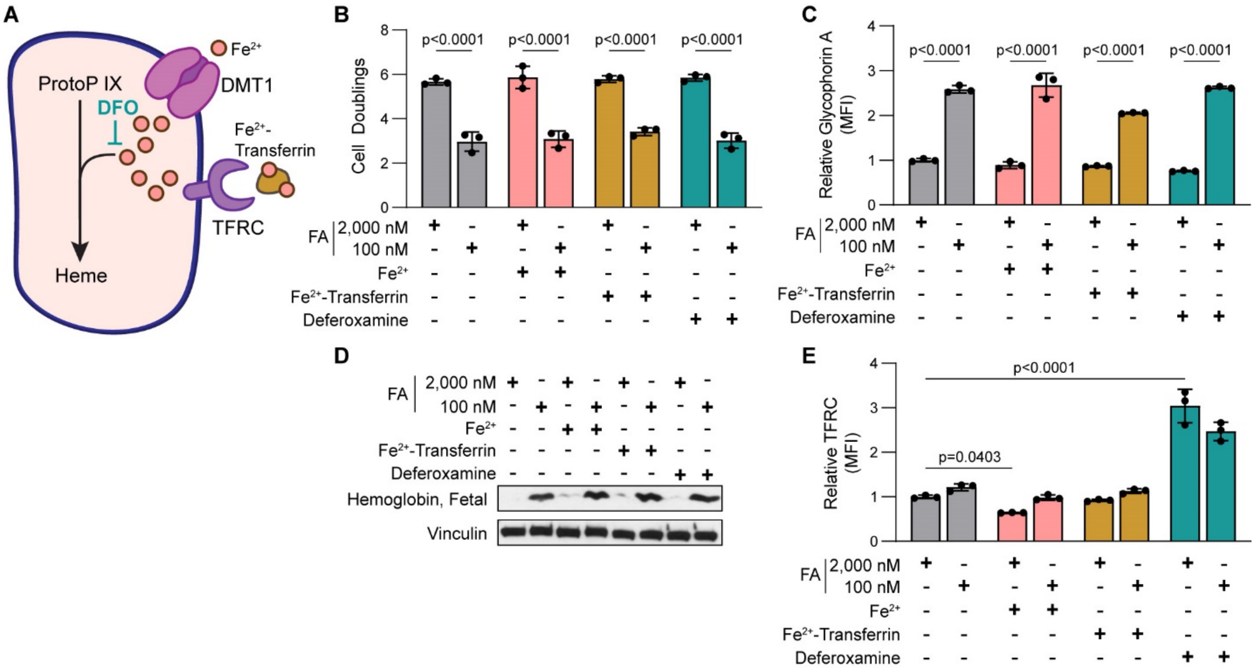

Supplementary Figure 2. No role for iron availability or limitation as a driver of folate-deprivation induced differentiation.

(A) Schematic depicting the role of iron intake and utilization in heme biosynthesis. **(B-E)** Cell proliferation (**B**), glycophorin A expression (**C**), hemoglobin expression (**D**), and transferrin receptor (TFRC) expression (**E**) in K562 following 6 days culture in 2,000 and 100 nM FA media supplemented with free iron (Ammonium Ferric Citrate), iron-bound transferrin, and the iron chelator, deferoxamine (DFO). Data shown are mean (± s.d.) of three biological replicates.

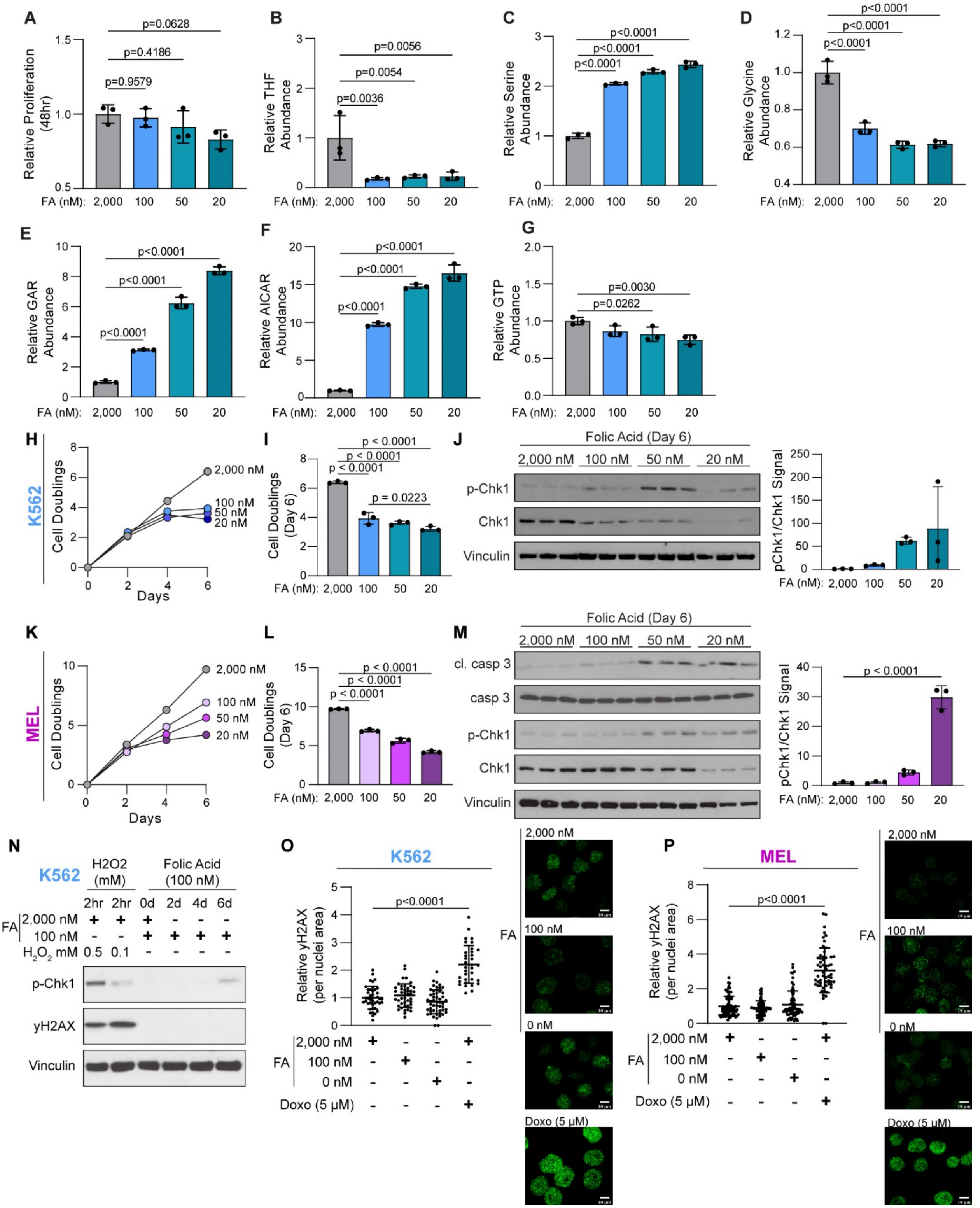

A
Relative Proliferation (48hr)
p=0.0628
p=0.4186
p=0.9579
FA (nM): 2,000 100 50 20
B
Relative THF Abundance
p=0.0056
p=0.0054
p=0.0036
FA (nM): 2,000 100 50 20
C
Relative Serine Abundance
p<0.0001
p<0.0001
p<0.0001
FA (nM): 2,000 100 50 20
D
Relative Glycine Abundance
p<0.0001
p<0.0001
p<0.0001
FA (nM): 2,000 100 50 20
E
Relative GAR Abundance
p<0.0001
p<0.0001
p<0.0001
FA (nM): 2,000 100 50 20
F
Relative AICAR Abundance
p<0.0001
p<0.0001
p<0.0001
FA (nM): 2,000 100 50 20
G
Relative GTP Abundance
p=0.0030
p=0.0262
FA (nM): 2,000 100 50 20
H
K562
Cell Doublings
Days
2,000 nM
100 nM
50 nM
20 nM
I
Cell Doublings (Day 6)
p < 0.0001
p < 0.0001
p < 0.0001
p = 0.0223
FA (nM): 2,000 100 50 20
J
Folic Acid (Day 6)
2,000 nM 100 nM 50 nM 20 nM
p-Chk1
Chk1
Vinculin
pChk1/Chk1 Signal
FA (nM): 2,000 100 50 20
K
MEL
Cell Doublings
Days
2,000 nM
100 nM
50 nM
20 nM
L
Cell Doublings (Day 6)
p < 0.0001
p < 0.0001
p < 0.0001
FA (nM): 2,000 100 50 20
M
Folic Acid (Day 6)
2,000 nM 100 nM 50 nM 20 nM
cl. casp 3
casp 3
p-Chk1
Chk1
Vinculin
pChk1/Chk1 Signal
p < 0.0001
FA (nM): 2,000 100 50 20
N
K562
H2O2 (mM)
Folic Acid (100 nM)
2hr 2hr 0d 2d 4d 6d
FA 2,000 nM + + + - - -
100 nM - - + + + +
H2O2 mM 0.5 0.1 - - - -
p-Chk1
yH2AX
Vinculin
O
K562
Relative yH2AX (per nuclei area)
p<0.0001
FA 2,000 nM + - - +
100 nM - + - -
0 nM - - + -
Doxo (5 µM) - - - +
2,000 nM
FA 100 nM
0 nM
Doxo (5 µM)
P
MEL
Relative yH2AX (per nuclei area)
p<0.0001
FA 2,000 nM + - - +
100 nM - + - -
0 nM - - + -
Doxo (5 µM) - - - +
2,000 nM
FA 100 nM
0 nM
Doxo (5 µM)

Supplementary Figure 3. DNA damage is induced by severe folate deprivation, but not following mild folate deprivation (100 nM).

(A, B) Cell proliferation of K562 over 48hr in the indicated concentration of folic acid. **(B-G)** Relative level of the indicated metabolite following 48hr culture of K562 in the indicated concentration of folic acid as measured by LC-MS. **(H, I)** Cell proliferation of K562 over 6 days in the indicated concentration of folic acid. **(J)** Western blotting for p-Chk1 following 6 days culture in the indicated folic acid concentration. Quantification is on the right. **(K, L)** Cell proliferation of MEL over 6 days in the indicated concentration of folic acid. **(M)** Western blotting for cleaved caspase 3 and p-Chk1 following 6 days culture in the indicated folic acid concentration (left) and quantification of p-Chk1/Chk1 western blotting signal (right). **(N)** Western blotting comparing p-Chk1 and yH2AX in K562 treated for 2 hr with H_2O_2 or up to 6 days with 100 nM FA. **(O, P)** Relative immunofluorescence yH2AX staining in **(O)** K562 and **(P)** MEL following 2-day culture in 2,000 nM, 100 nM, or 0 nM FA. Doxorubicin (Doxo, 2hr) served as a positive control for DNA damage. Scale bar = 10 μM. Each dot represents a unique cell. Data shown are mean (± s.d.) of three biological replicates.

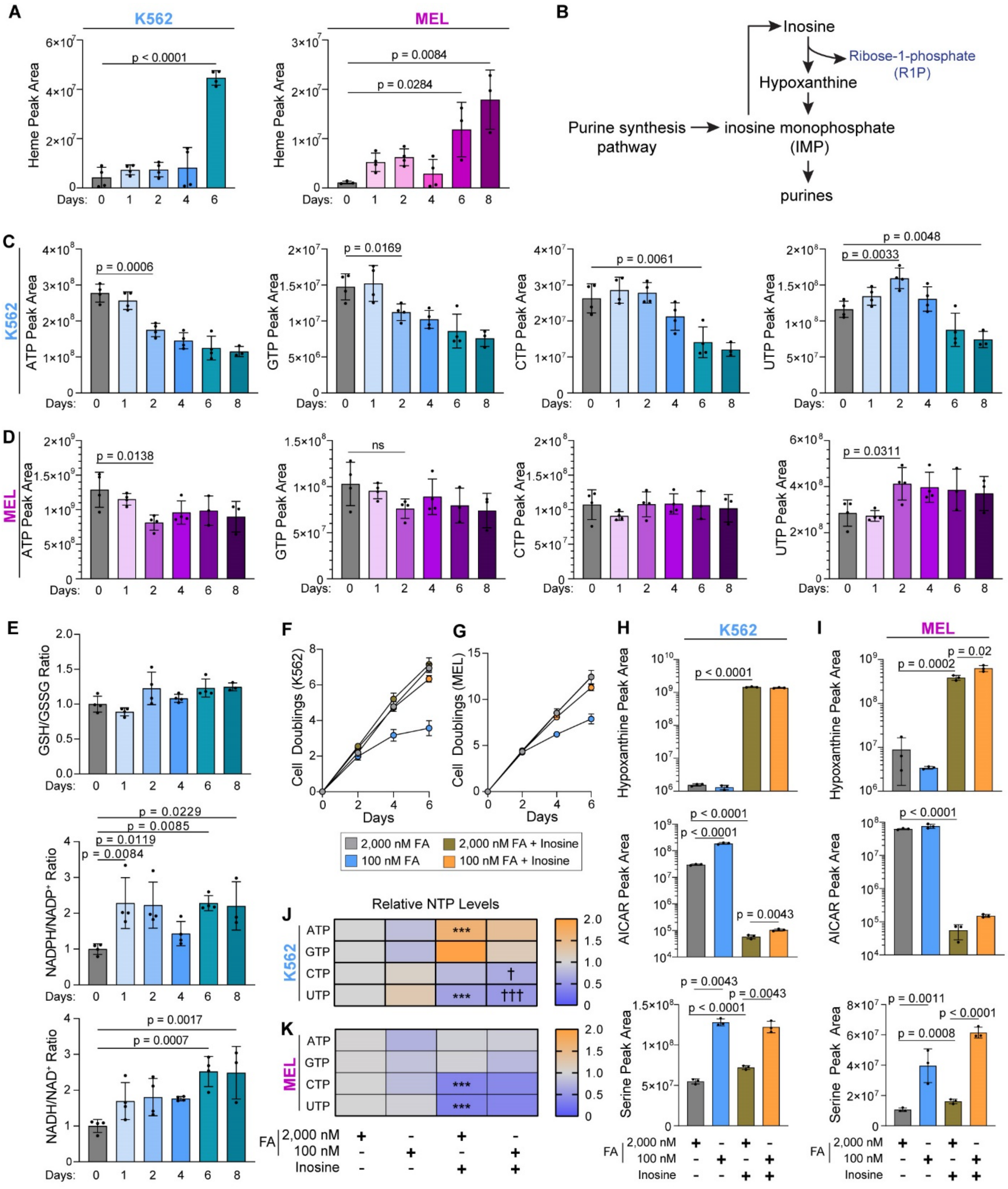

A
K562
Heme Peak Area
p < 0.0001
Days: 0 1 2 4 6
MEL
p = 0.0084
p = 0.0284
Days: 0 1 2 4 6 8
B
Inosine
Ribose-1-phosphate
(R1P)
Hypoxanthine
Purine synthesis
pathway
inosine monophosphate
(IMP)
purines
C
K562
ATP Peak Area
p = 0.0006
GTP Peak Area
p = 0.0169
CTP Peak Area
p = 0.0061
UTP Peak Area
p = 0.0048
p = 0.0033
Days: 0 1 2 4 6 8
D
MEL
ATP Peak Area
p = 0.0138
GTP Peak Area
ns
CTP Peak Area
UTP Peak Area
p = 0.0311
Days: 0 1 2 4 6 8
E
GSH/GSSG Ratio
Days: 0 1 2 4 6 8
NADPH/NADP+ Ratio
p = 0.0229
p = 0.0085
p = 0.0119
p = 0.0084
NADH/NAD+ Ratio
p = 0.0017
p = 0.0007
F
Cell Doublings (K562)
Days
G
Cell Doublings (MEL)
Days
2,000 nM FA
100 nM FA
2,000 nM FA + Inosine
100 nM FA + Inosine
H
K562
Hypoxanthine Peak Area
p < 0.0001
AICAR Peak Area
p < 0.0001
p < 0.0001
p = 0.0043
Serine Peak Area
p = 0.0043
p < 0.0001
p = 0.0043
I
MEL
Hypoxanthine Peak Area
p = 0.0002
p = 0.02
AICAR Peak Area
p < 0.0001
Serine Peak Area
p = 0.0011
p = 0.0008
p < 0.0001
J
Relative NTP Levels
K562
ATP
GTP
CTP
UTP
***
†
†††
K
MEL
***
FA 2,000 nM + - + -
100 nM - + - +
Inosine - - + +

Supplementary Figure 4. Purine synthesis inhibition, and not pyrimidine synthesis, nucleotide imbalance, or oxidative stress, is observed in the early days following folate deprivation.

(A) Heme levels in K562 (top) and MEL (bottom) through day 8 in 100 nM FA as measured by LC-MS. **(B)** Simplified schematic depicting the purine synthesis and salvage pathways. **(C, D)** Nucleotide triphosphate levels in K562 (**C**) and MEL (**D**) measured by LC-MS at day 8 in 100 nM FA. **(E)** Redox metabolite levels in K562 measured by LC-MS over 8 days in 100 nM FA. Top: The ratio of glutathione (GSH) to oxidized glutathione (GSSG). Middle: The ratio of NADPH to NADP+. Bottom: The ratio of NADH to NAD+. **(F, G)** Proliferation of K562 (**F**) and MEL (**G**) over 6 days in 2,000 and 100 nM FA alone, or with inosine supplementation. **(H, I)** Hypoxanthine, AICAR, and Serine levels in K562 (**H**) and MEL (**I**) following 2 days in 2,000 and 100 nM FA with or without inosine supplementation. **(J, K)** Relative levels of nucleotide triphosphate after 2 days in 2,000 and 100 nM FA, with or without inosine supplementation in K562 (**J**) and MEL (**K**). * - significance between 2,000 nM FA vs 2,000 nM FA+inosine. † - significance between 100 nM FA vs 100 nM FA+inosine. Data shown are mean (± s.d.) of three biological replicates.

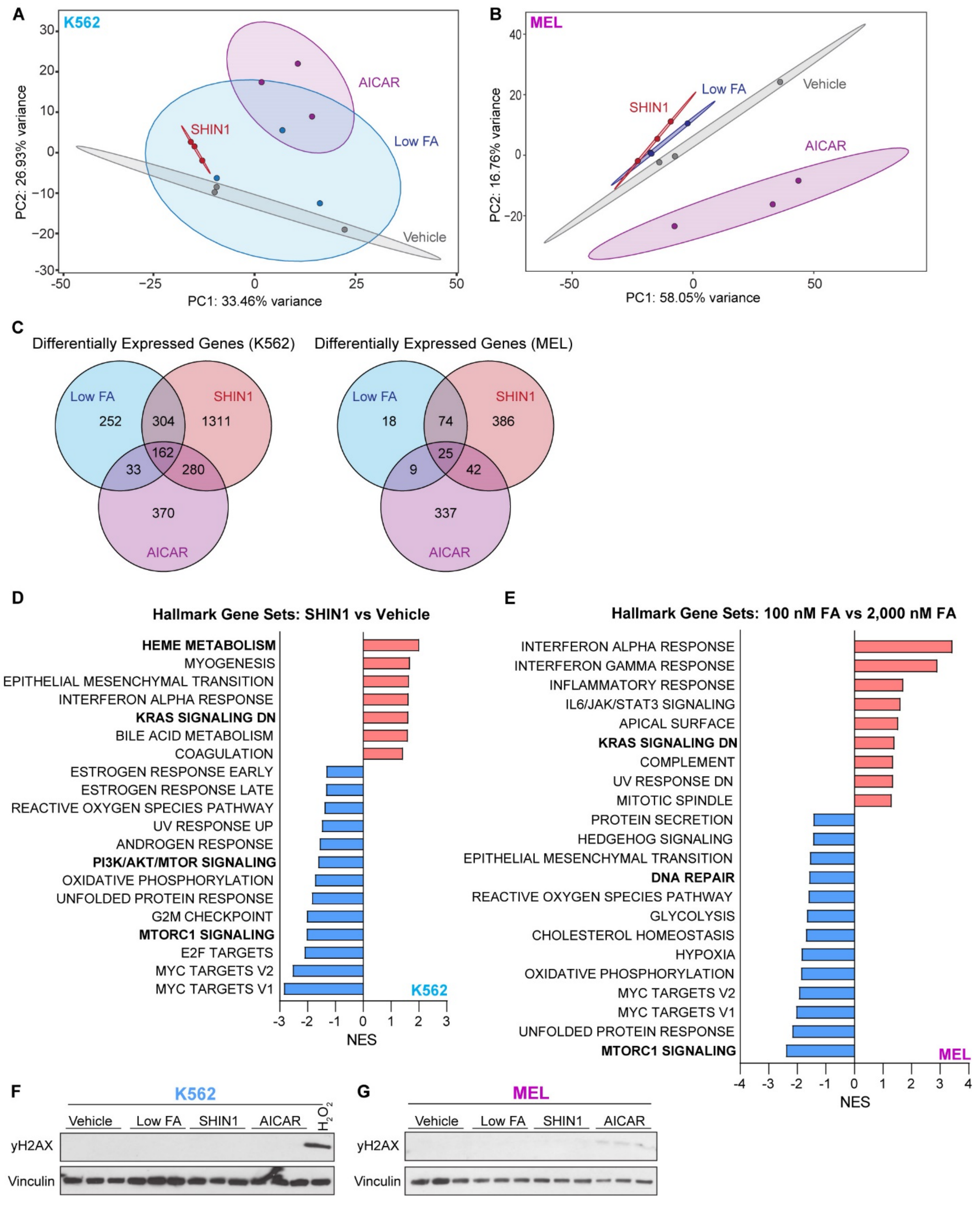
A
K562
AICAR
Low FA
SHIN1
Vehicle
PC1: 33.46% variance
PC2: 26.93% variance
B
MEL
Low FA
Vehicle
SHIN1
AICAR
PC1: 58.05% variance
PC2: 16.76% variance
C
Differentially Expressed Genes (K562)
Low FA
SHIN1
252
304
1311
162
33
280
370
AICAR
Differentially Expressed Genes (MEL)
Low FA
SHIN1
18
74
386
25
9
42
337
AICAR
D
Hallmark Gene Sets: SHIN1 vs Vehicle
HEME METABOLISM
MYOGENESIS
EPITHELIAL MESENCHYMAL TRANSITION
INTERFERON ALPHA RESPONSE
KRAS SIGNALING DN
BILE ACID METABOLISM
COAGULATION
ESTROGEN RESPONSE EARLY
ESTROGEN RESPONSE LATE
REACTIVE OXYGEN SPECIES PATHWAY
UV RESPONSE UP
ANDROGEN RESPONSE
PI3K/AKT/MTOR SIGNALING
OXIDATIVE PHOSPHORYLATION
UNFOLDED PROTEIN RESPONSE
G2M CHECKPOINT
MTORC1 SIGNALING
E2F TARGETS
MYC TARGETS V2
MYC TARGETS V1
K562
NES
E
Hallmark Gene Sets: 100 nM FA vs 2,000 nM FA
INTERFERON ALPHA RESPONSE
INTERFERON GAMMA RESPONSE
INFLAMMATORY RESPONSE
IL6/JAK/STAT3 SIGNALING
APICAL SURFACE
KRAS SIGNALING DN
COMPLEMENT
UV RESPONSE DN
MITOTIC SPINDLE
PROTEIN SECRETION
HEDGEHOG SIGNALING
EPITHELIAL MESENCHYMAL TRANSITION
DNA REPAIR
REACTIVE OXYGEN SPECIES PATHWAY
GLYCOLYSIS
CHOLESTEROL HOMEOSTASIS
HYPOXIA
OXIDATIVE PHOSPHORYLATION
MYC TARGETS V2
MYC TARGETS V1
UNFOLDED PROTEIN RESPONSE
MTORC1 SIGNALING
MEL
NES
F
K562
Vehicle
Low FA
SHIN1
AICAR
H2O2
yH2AX
Vinculin
G
MEL
Vehicle
Low FA
SHIN1
AICAR
yH2AX
Vinculin

Supplementary Figure 5. Gene expression analysis in erythroid cells following perturbation of one-carbon metabolism.

(A, B) Principal component analysis (PCA) clustering of vehicle (2,000 nM FA), Low FA (100 nM FA), SHIN1, or AICAR treated **(A)** K562 or **(B)** MEL based on RNA sequencing analysis following 3 days in culture. **(C)** The overlapping differentially expressed genes in K562 and MEL following vehicle (2,000 nM FA), Low FA (100 nM FA), SHIN1, or AICAR treatment. **(D, E)** Geneset Enrichment Analysis (GSEA) using the Hallmarks genesets on **(D)** K562 treated with SHIN1 compared to vehicle and **(E)** MEL treated with 100 nM FA compared to 2000 nM FA. Positively enriched genesets are denoted in pink. Negatively associated genesets are denoted in blue. **(F, G)** Western blotting for yH2AX in **(F)** K562 and **(G)** MEL following treatment with vehicle (2,000 nM FA), Low FA (100 nM FA), SHIN1 (1.25 μM), or AICAR (500 nM). Data shown are mean (± s.d.) of three biological replicates.

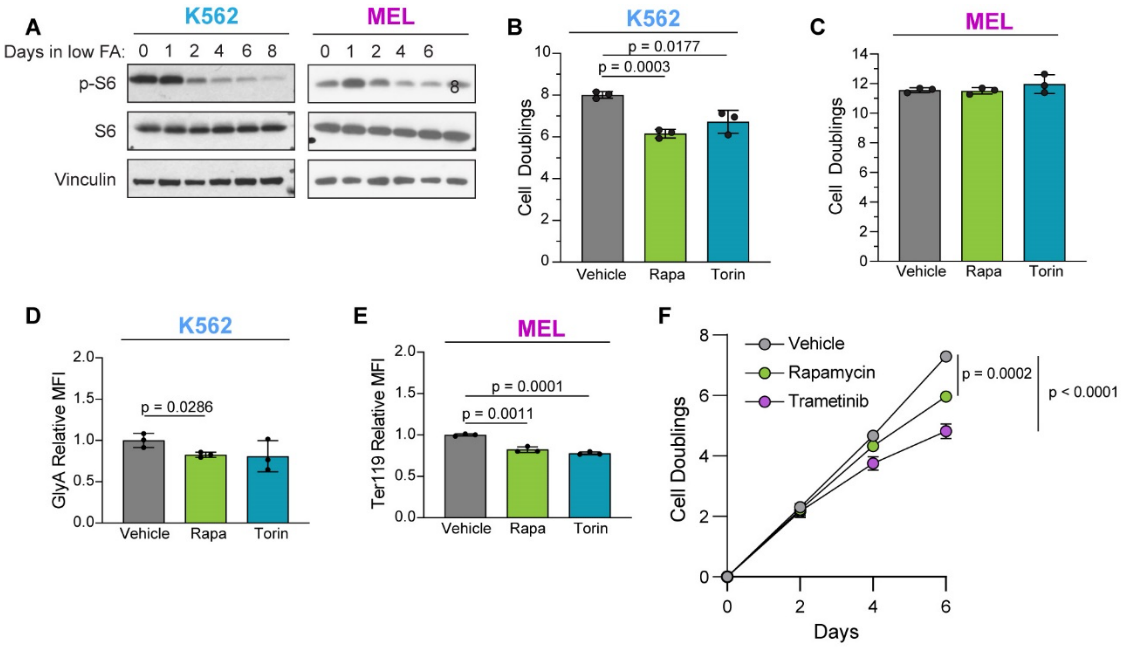

Supplementary Figure 6. mTORC1 inactivation is not necessary or sufficient to induce erythroid differentiation.
(A) Western blot analysis of phospho-S6 and total S6 levels in K562 and MEL over 8 days in 100 nM FA. Vinculin is a loading control. **(B, C)** Cell proliferation of K562 (**B**) and MEL (**C**) at day 6 in vehicle, Rapamycin (100 µM), and Torin1 (5 nM) treatments. Media and drug treatments were refreshed every 2 days. **(D)** Cell surface Glycophorin A levels on vehicle-, Rapamycin-, and Torin1-treated K562 (for 6 days). **(E)** Cell surface Ter119 levels on vehicle-, Rapamycin-, and Torin1-treated MEL (for 6 days). **(F)** Cell proliferation of K652 treated with Rapamycin (100 µM) or Trametinib (30 nM) over 6 days. Data shown are mean (± s.d.) of three biological replicates.

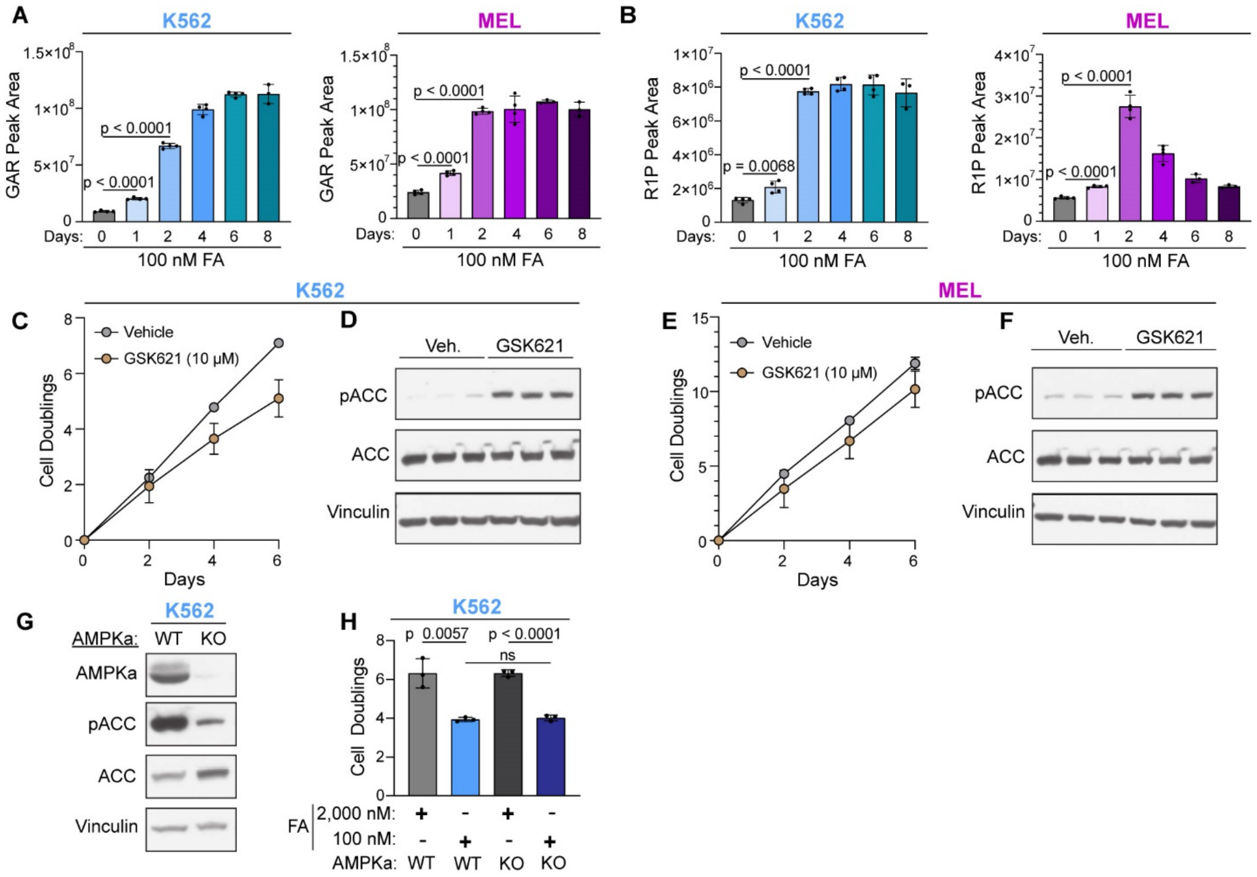

Supplementary Figure 7. Purine metabolites in folate-deprived erythroid cells and AMPK pharmacologic activation and genetic inhibition.

(A) GAR levels in K562 (left) and MEL (right) over 8 days in 100 nM FA measured by LC-MS. **(B)** Ribose-1-phosphate (R1P) levels in K562 (left) and MEL (right) over 8 days in 100 nM FA measured by LC-MS. **(C, E)** Proliferation of vehicle- and GSK621-treated (10 µM) K562 (**C**) and MEL (**E**) (for 6 days). **(D, F)** Western blot analysis of phospho-ACC/ACC, with and without GSK621 treatment in K562 (**D**) and MEL (**F**). Vinculin is a loading control. **(G)** Western blot analysis of AMPKa1/a2 WT and DKO K562. Vinculin is a loading control. **(H)** Proliferation of AMPKa1/a2 WT and DKO K562 in 2,000 and 100 nM FA over 6 days. Data shown are mean (± s.d.) of three biological replicates.

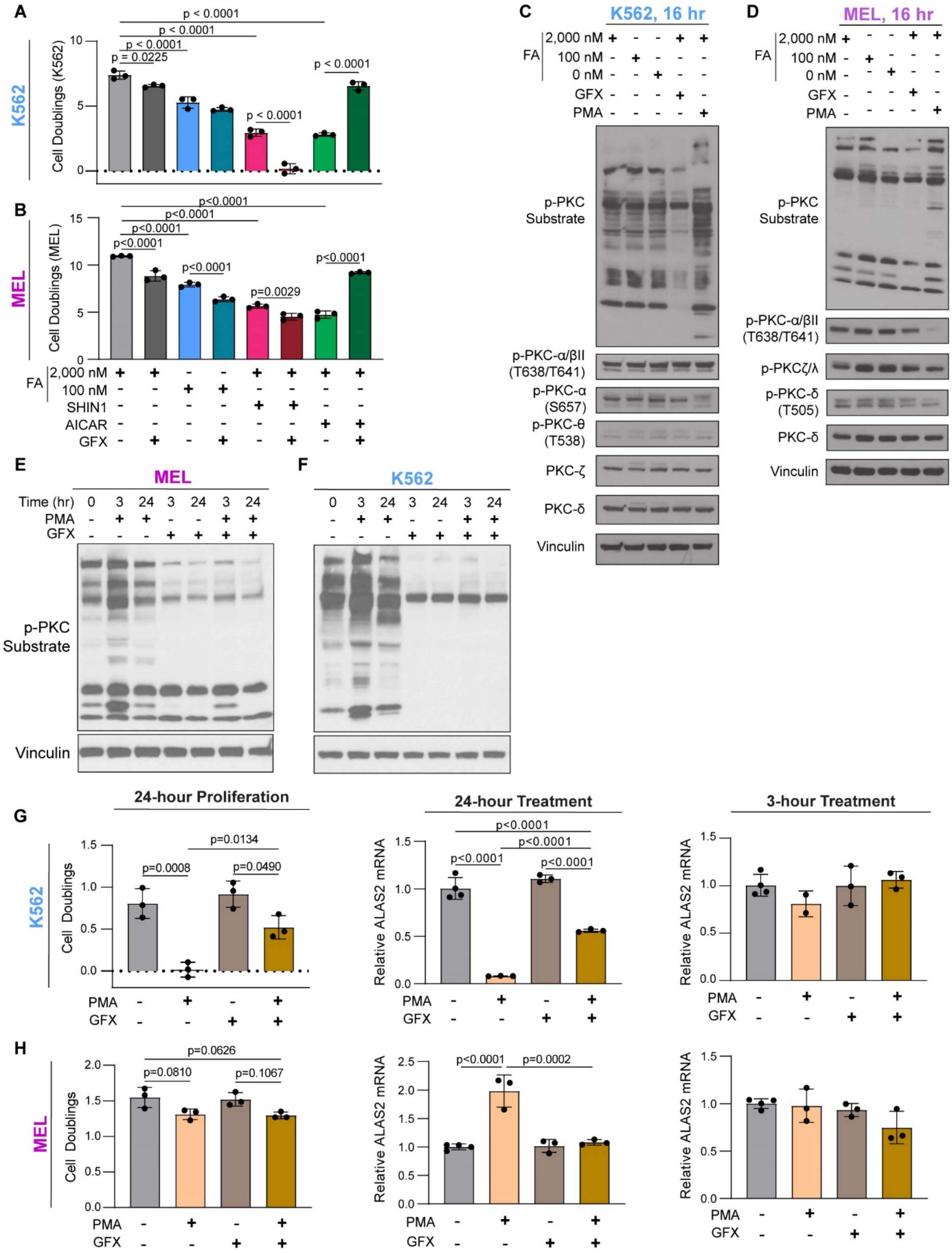
A
K562
Cell Doublings (K562)
p < 0.0001
p = 0.0225
p < 0.0001
p < 0.0001
p < 0.0001
p < 0.0001
B
MEL
Cell Doublings (MEL)
p<0.0001
p<0.0001
p<0.0001
p<0.0001
p<0.0001
p=0.0029
p<0.0001
FA
2,000 nM
100 nM
SHIN1
AICAR
GFX
C
K562, 16 hr
D
MEL, 16 hr
FA
2,000 nM
100 nM
0 nM
GFX
PMA
p-PKC Substrate
p-PKC-α/βII (T638/T641)
p-PKC-α (S657)
p-PKC-θ (T538)
PKC-ζ
PKC-δ
Vinculin
p-PKCζ/λ
p-PKC-δ (T505)
E
MEL
F
K562
Time (hr)
PMA
GFX
p-PKC Substrate
Vinculin
G
K562
24-hour Proliferation
Cell Doublings
p=0.0008
p=0.0134
p=0.0490
24-hour Treatment
Relative ALAS2 mRNA
p<0.0001
p<0.0001
p<0.0001
p<0.0001
3-hour Treatment
Relative ALAS2 mRNA
PMA
GFX
H
MEL
Cell Doublings
p=0.0626
p=0.0810
p=0.1067
Relative ALAS2 mRNA
p<0.0001
p=0.0002
Relative ALAS2 mRNA
PMA
GFX

Supplementary Figure 8. PKC's role in folate deprivation-induced erythroid differentiation.

(A) Cell proliferation over 6 days in K562 following Low FA, SHIN1 (1.25 μM), or AICAR (500 nM) treatment plus PKC inhibition with the PKC inhibitor, GF109203X (GFX, 5uM) **(B)** Cell proliferation over 6 days in MEL following Low FA, SHIN1 (1.25 μM), or AICAR (500 nM) treatment plus PKC inhibition with the PKC inhibitor, GF109203X (GFX, 5uM). **(C, D)** Western blot analysis of PKC signaling following 16 hr treatment with the indicated FA concentration, with GFX and PMA as PKC signaling controls in **(C)** K562 and **(D)** MEL. **(E, F)** Western blot analysis of PKC signaling following 3- and 24-hr treatment with PMA (100ng/mL), GFX (5 μM), or the combination in **(E)** K562 and **(F)** MEL. **(G)** Cell proliferation over 24-hr treatment and ALAS2 mRNA expression over 3- and 24- hr treatment with PMA (100ng/mL), GFX (5 μM), or the combination in K562. **(H)** Cell proliferation over 24-hr treatment and ALAS2 mRNA expression over 3- and 24- hr treatment with PMA (100ng/mL), GFX (5 μM), or the combination in MEL. Data shown are mean (± s.d.) of three biological replicates.

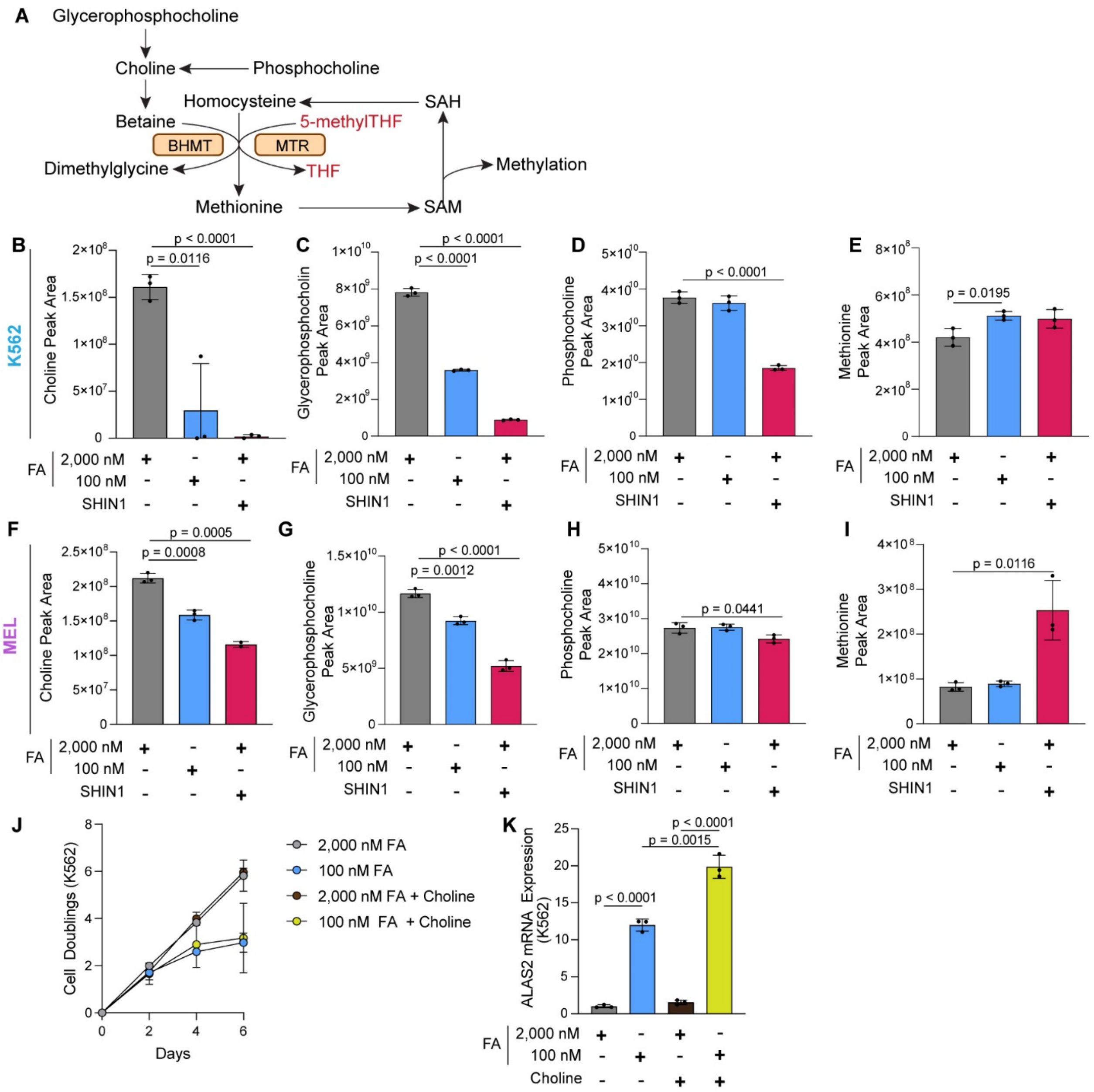

Supplementary Figure 9. Folate deprivation depletes choline metabolism intermediates, but choline supplementation is not sufficient to rescue folate-depletion induced differentiation Folate deprivation depletes choline metabolism intermediates, but choline supplementation is not sufficient to rescue folate-depletion induced differentiation.

(A) Schematic depicting the shared reaction between choline and folate metabolism. **(B-E)** Indicated metabolite levels in K562 cultured for 2 days in 2,000 nM FA, 100 nM FA, and 2,000 nM FA + SHIN1 measured by LC-MS. **(F-I)** Indicated metabolite levels measured by LC-MS in MEL in the same conditions as **B-E.** **(J)** Cell Proliferation of K562 in 2,000 and 100 nM FA with or without supplementation with choline (500 µM). **(K)** RT-qPCR analysis of ALAS2 mRNA in choline-supplemented K562 cells following 6 days in 2,000 and 100 nM FA. Data shown are mean (± s.d.) of three biological replicates.

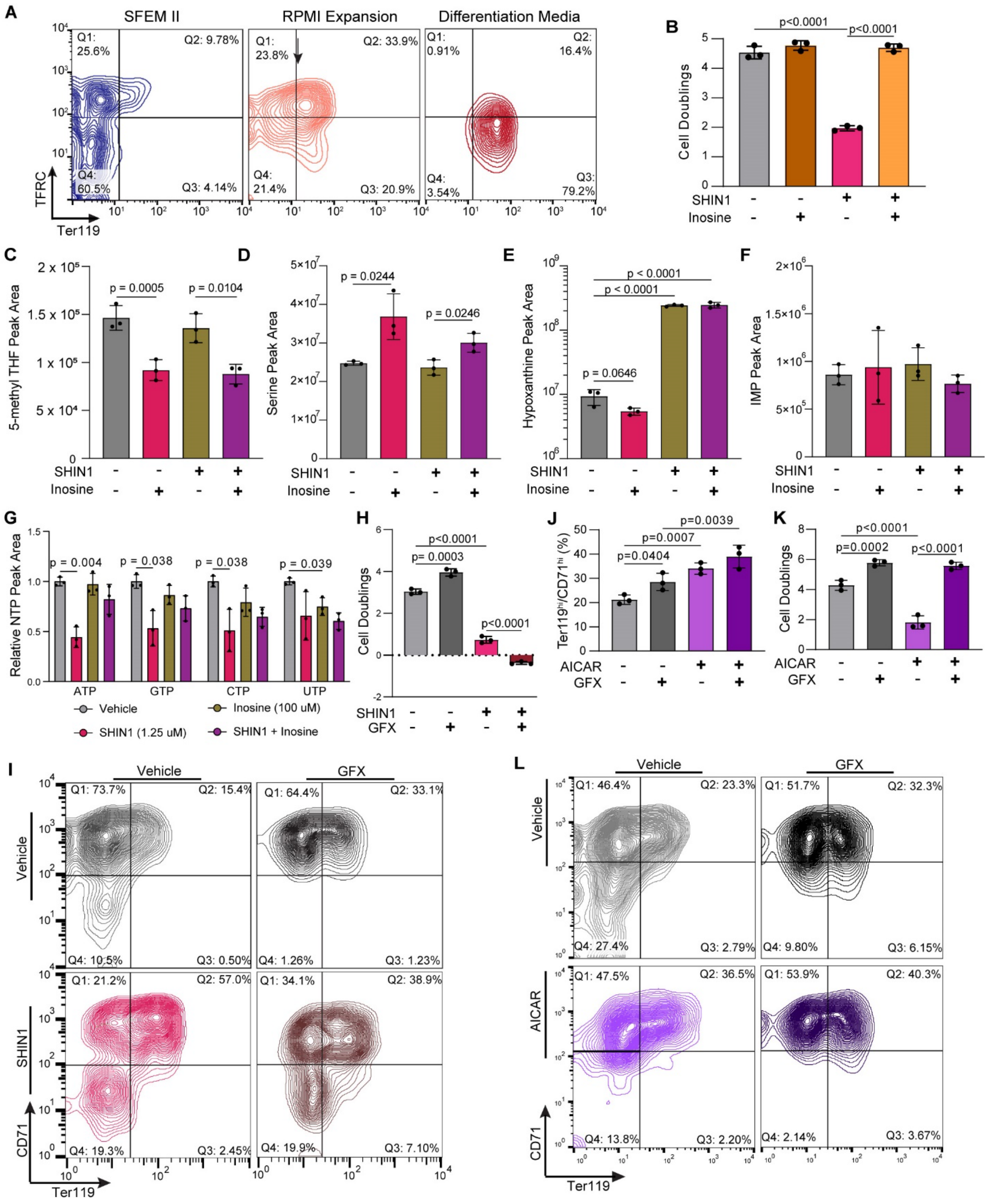

A
SFEM II
RPMI Expansion
Differentiation Media
Q1: 25.6%
Q2: 9.78%
Q3: 4.14%
Q4: 60.5%
Q1: 23.8%
Q2: 33.9%
Q3: 20.9%
Q4: 21.4%
Q1: 0.91%
Q2: 16.4%
Q3: 79.2%
Q4: 3.54%
TFRC
Ter119
B
Cell Doublings
p<0.0001
p<0.0001
SHIN1
Inosine
C
5-methyl THF Peak Area
p = 0.0005
p = 0.0104
D
Serine Peak Area
p = 0.0244
p = 0.0246
E
Hypoxanthine Peak Area
p < 0.0001
p < 0.0001
p = 0.0646
F
IMP Peak Area
G
Relative NTP Peak Area
p = 0.004
p = 0.038
p = 0.038
p = 0.039
ATP
GTP
CTP
UTP
Vehicle
Inosine (100 uM)
SHIN1 (1.25 uM)
SHIN1 + Inosine
H
Cell Doublings
p<0.0001
p= 0.0003
p<0.0001
SHIN1
GFX
J
Ter119hi/CD71hi (%)
p=0.0039
p=0.0007
p=0.0404
AICAR
GFX
K
Cell Doublings
p<0.0001
p=0.0002
p<0.0001
AICAR
GFX
I
Vehicle
GFX
Vehicle
SHIN1
Q1: 73.7%
Q2: 15.4%
Q3: 0.50%
Q4: 10.5%
Q1: 64.4%
Q2: 33.1%
Q3: 1.23%
Q4: 1.26%
Q1: 21.2%
Q2: 57.0%
Q3: 2.45%
Q4: 19.3%
Q1: 34.1%
Q2: 38.9%
Q3: 7.10%
Q4: 19.9%
CD71
Ter119
L
Vehicle
GFX
Vehicle
AICAR
Q1: 46.4%
Q2: 23.3%
Q3: 2.79%
Q4: 27.4%
Q1: 51.7%
Q2: 32.3%
Q3: 6.15%
Q4: 9.80%
Q1: 47.5%
Q2: 36.5%
Q3: 2.20%
Q4: 13.8%
Q1: 53.9%
Q2: 40.3%
Q3: 3.67%
Q4: 2.14%
CD71
Ter119

Supplementary Figure 10. Folate deprivation induces differentiation in murine primary erythroid progenitor cells.

(A) Representative flow cytometry plots depicting Ter119 and CD71 (TFRC) expression following 4 days culture of isolated erythroid progenitor cells in SFEM II expansion media, our custom, RPMI-based expansion media, or in erythroid differentiation media. (B) Proliferation of vehicle-, SHIN1 (1.25 μM)-, inosine (100 μM)-, or SHIN1 + inosine-treated murine primary erythroid progenitor cells following 4-days culture. Cells were cultured in SFEM II expansion media. (C-G) Indicated metabolite abundance as measured by LC-MS in murine primary erythroid progenitor cells cultured in SFEM II expansion media supplemented with vehicle-, SHIN1 (1.25 μM)-, inosine (100 μM)-, or SHIN1 + inosine for 2 days. (H) Proliferation of vehicle, SHIN1 (1.25 μM), GFX (5 μM), or SHIN1+GFX treated murine primary erythroid progenitor cells following 4-days culture in SFEM II expansion media. (I) Representative flow cytometry plots of vehicle, SHIN1 (1.25 μM), GFX (5 μM), or SHIN1+GFX treated murine primary erythroid progenitor cells following 4-days culture in SFEM II expansion media. (J) Proliferation of vehicle, AICAR (500 nM), GFX (5 μM), or AICAR+GFX treated murine primary erythroid progenitor cells following 4-days culture in SFEM II expansion media. (K) Representative flow cytometry plots of vehicle, AICAR (500 nM), GFX (5 μM), or AICAR+GFX treated murine primary erythroid progenitor cells following 4-days culture in SFEM II expansion media. Data shown are mean (± s.d.) of three biological replicates. (L) Representative flow cytometry plots of vehicle, AICAR (500 nM), GFX (5 μM), or AICAR+GFX treated murine primary erythroid progenitor cells following 4-days culture in SFEM II expansion media.

Supplementary data files:

Data S1 – This file contains the data points used to generate each of the figures in the paper.
Data S2 – This file contains list of metabolomics datasets deposited to Metabolomics Work Bench.
Data S3 – This file contains analyzed RNAseq data.
